# Comprehensive Genomic Investigation of Adaptive Mutations Driving the Low-Level Oxacillin Resistance Phenotype in Staphylococcus aureus

**DOI:** 10.1128/mBio.02882-20

**Published:** 2020-12-08

**Authors:** Stefano G. Giulieri, Romain Guérillot, Jason C. Kwong, Ian R. Monk, Ashleigh S. Hayes, Diane Daniel, Sarah Baines, Norelle L. Sherry, Natasha E. Holmes, Peter Ward, Wei Gao, Torsten Seemann, Timothy P. Stinear, Benjamin P. Howden

**Affiliations:** a Department of Microbiology and Immunology, The University of Melbourne at the Doherty Institute for Infection and Immunity, Melbourne, Australia; b Department of Infectious Diseases, Austin Health, Heidelberg, Australia; c Department of Microbiology, Austin Health, Heidelberg, Australia; d Microbiological Diagnostic Unit Public Health Laboratory, The University of Melbourne at the Doherty Institute for Infection and Immunity, Melbourne, Australia; Yale School of Public Health

**Keywords:** *Staphylococcus aureus*, antibiotic resistance, β-lactams, genomics

## Abstract

Antistaphylococcal penicillins such as oxacillin are the key antibiotics in the treatment of invasive methicillin-susceptible Staphylococcus aureus (MSSA) infections; however, *mec* gene-independent resistance adaptation can cause treatment failure. Despite its clinical relevance, the basis of this phenomenon remains poorly understood. Here, we investigated the genomic adaptation to oxacillin at an unprecedented scale using a large collection of 503 clinical *mec*-negative isolates and 30 *in vitro*-adapted isolates from independent oxacillin exposures. By combining comparative genomics, evolutionary convergence, and genome-wide association analysis, we found 21 genetic loci associated with low-level oxacillin resistance, underscoring the polygenic nature of this phenotype. Evidence of adaptation was particularly strong for the c-di-AMP signal transduction pathways (*gdpP* and *dacA*) and in the *clpXP* chaperone-protease complex. The role of mutations in *gdpP* in conferring low-level oxacillin resistance was confirmed by allele-swapping experiments. We found that resistance to oxacillin emerges at high frequency *in vitro* (median, 2.9 × 10^−6^; interquartile range [IQR], 1.9 × 10^−6^ to 3.9 × 10^−6^), which is consistent with a recurrent minimum inhibitory concentration (MIC) increase across the global phylogeny of clinical isolates. Nevertheless, adaptation in clinical isolates appears sporadically, with no stably adapted lineages, suggesting a high fitness cost of resistance, confirmed by growth assessment of mutants in rich media. Our data provide a broader understanding of the emergence and dynamics of oxacillin resistance adaptation in S. aureus and a framework for future surveillance of this clinically important phenomenon.

## Abstract (precis)

The majority of Staphylococcus aureus strains causing human disease are methicillin-susceptible (MSSA) and can be treated with antistaphylococcal penicillins (such as oxacillin). While acquisition of the *mec* gene represents the main resistance mechanism to oxacillin, S. aureus can acquire low-level resistance through adaptive mutations in other genes. In this study, we used genomic approaches to understand the basis of S. aureus adaption to oxacillin and its dynamic at the population level. By combining a genome analysis of clinical isolates from persistent MSSA infections, *in vitro* selection of oxacillin resistance, and genome-wide association analysis on a large collection of isolates, we identified 21 genes linked to secondary oxacillin resistance. Adaptive mutations in these genes were easy to select when S. aureus was exposed to oxacillin, but they also came at a substantial cost in terms of bacterial fitness, suggesting that this phenotype emerges preferentially in the setting of sustained antibiotic exposure.

## INTRODUCTION

Staphylococcus aureus is a major cause of severe infections, most notably bloodstream infections, infective endocarditis, and osteoarticular infections (1). The clinical outcomes of S. aureus infections are a result of a complex interplay between host, treatment, and pathogen (2). Among them, methicillin resistance (mediated by penicillin-binding protein 2a [PBP2a] and encoded by the *mec* gene) has been associated with higher mortality, persistent infection, and treatment failure (3). However, outcomes of methicillin-susceptible S. aureus (MSSA) infections can also be severe. For example, mortality of MSSA bacteremia has been estimated at 15 to 20% (4). Moreover, studies show that while the incidence of methicillin-resistant S. aureus (MRSA) is decreasing, MSSA infections appear to be on the rise (5, 6). Since MSSA infections contribute overwhelmingly to the burden of invasive S. aureus infections (up to 95% in some reports [5]), it is important to identify factors associated with treatment failure with antistaphylococcal penicillins (oxacillin, flucloxacillin, and nafcillin), the first-line treatment for invasive MSSA.

PPB2a-independent oxacillin resistance can lead to treatment failure in MSSA infections, and these isolates typically exhibit an oxacillin minimum inhibitory concentration (MIC) of between 2 and 16 mg/liter (7). Until recently, two distinct hypotheses were invoked to explain the occurrence of low-level oxacillin resistance. Studies from the 1980s pointed to hyperproduction of β-lactamase (8). Strains exhibiting this phenotype were referred to as borderline oxacillin-resistant S. aureus (BORSA) (9). Alternatively, point mutations in penicillin-binding proteins were identified in oxacillin-resistant isolates and confirmed by complementation experiments in the oxacillin-sensitive strain RN6390 (10). This mechanism is analogous to penicillin resistance in pneumococci (11) and similar to ceftaroline resistance in S. aureus (12, 13). The term MODSA (modified PBP S. aureus) is used to label these isolates (14). However, recent studies support the role of alternative adaptive pathways, such as those involving *gdpP* in low-level oxacillin resistance (15, 16). These findings indicate that the mutational landscape of non-*mec*-mediated low-level oxacillin resistance is more complex than previously thought and requires further exploration.

To comprehensively explore genetic changes associated with this phenotype, we undertook a multifaceted comparative genomics study, including whole-genome sequencing (WGS) of 13 isolates from two clinical cases of persistent MSSA infection, combined with WGS of 30 *in vitro*-selected oxacillin-adapted mutants and a genome-wide association study (GWAS) approach using 490 isolates. This analysis allowed us to map adaptive mutations arising under the selective pressure of oxacillin and to define a distinctive phenotype (characterized by low-level oxacillin resistance, impaired growth fitness, and sporadic occurrence) that has the potential to explain treatment failure in MSSA infections. To capture this phenotype and distinguish it from MRSA, we propose the new term “*mec*-independent oxacillin-nonsusceptible S. aureus” (MIONSA).

## RESULTS

### Comparative genomics of S. aureus isolates from two cases of flucloxacillin treatment failure with secondary low-level resistance.

**FIG 1:**
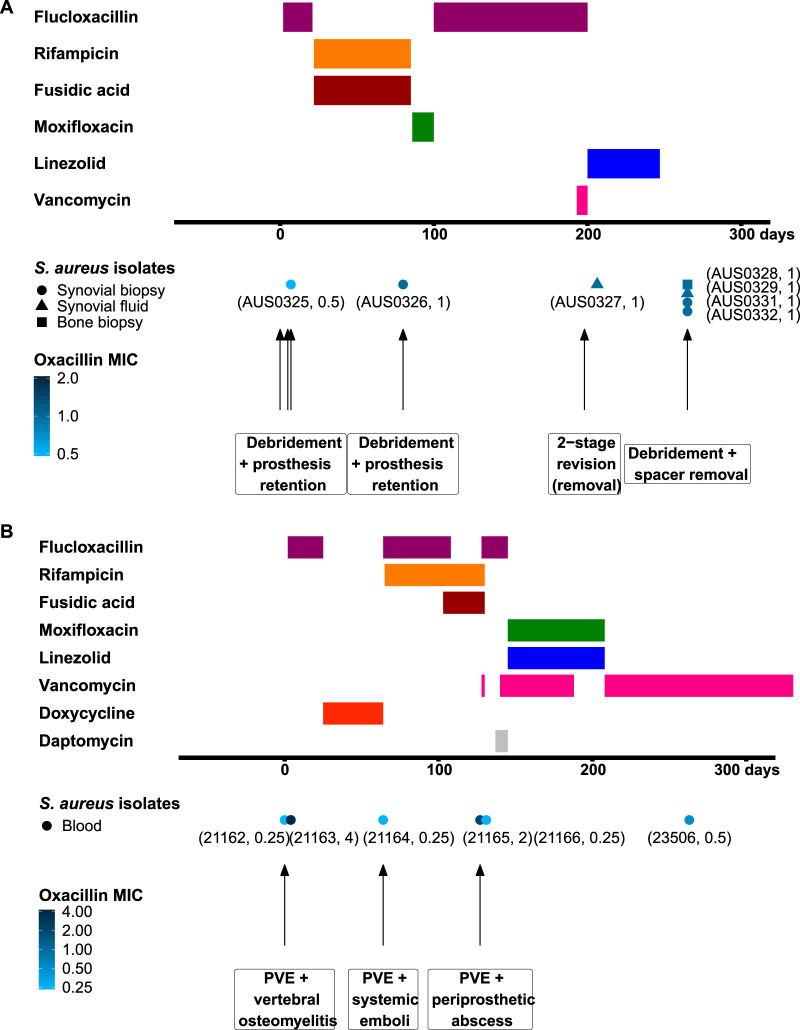
Overview of two clinical cases of persistent MSSA infection. Shown is the timeline (days) of surgical interventions (case 1 in panel A) or clinical events (case 2 in panel B), antibiotic treatments, oxacillin MIC and susceptibility testing of case 1, a persistent prosthetic knee infection (upper panel), and case 2 (lower panel), a relapsing prosthetic valve endocarditis (PVE) associated with pacemaker infection. The isolate name and oxacillin MIC (also depicted by the color scale) are reported in parentheses next to the symbols representing the clinical samples. The oxacillin MIC is in mg/liter.

Two cases of persistent device-associated MSSA infection with failure of flucloxacillin treatment and secondary MIC increase during treatment were identified. Case 1 was a patient with prosthetic knee infection that persisted for several months despite multiple surgical interventions and multiple courses of combination antibiotic treatment, including flucloxacillin, rifampin, and fusidic acid. S. aureus isolates collected during sequential surgeries displayed a progressive increase in oxacillin MICs from 0.5 to 1 mg/liter, as well as secondary resistance to rifampin. Case 2 was a patient with prosthetic valve and pacemaker-associated endocarditis that recurred twice after ceasing flucloxacillin-based antibiotic treatments. The baseline MIC for oxacillin was 0.25 mg/liter, but it increased to 4 mg/liter in S. aureus blood isolates recovered during the first flucloxacillin treatment course and upon the second recurrence of endocarditis. (The latter was also associated with secondary resistance to rifampin and fusidic acid.) Timing of clinical events, antimicrobial treatments, and microbiological features of the two cases are shown in Table 1 and Fig. 1.

**TABLE 1:** Microbiological features of 13 clinical isolates from 2 cases of flucloxacillin treatment failure with secondary oxacillin resistance

Clinical case (diagnosis)	Isolate ID	Source	Time post-initial infection (days)	Oxacillin MIC (mg/liter)	Other resistance phenotype(s)	MLST	Resistance gene
Case 1 (prosthetic-joint infection)	AUS0325	Synovial biopsy	0	0.5		88	*blaZ*
	AUS0326	Synovial biopsy	73	1	Rifampin	88	*blaZ*
	AUS0327	Synovial fluid	187	1	Rifampin	88	*blaZ*
	AUS0328	Bone biopsy	258	1	Rifampin	88	*blaZ*
	AUS0329	Synovial fluid	258	1	Rifampin	88	*blaZ*
	AUS0331	Synovial biopsy	258	1	Rifampin	88	*blaZ*
	AUS0332	Synovial biopsy	258	1	Rifampin	88	*blaZ*

Case 2 (prosthetic-valve endocarditis)	21162	Blood	0	0.25		34	*blaZ*
	21163	Blood	4	4		34	*blaZ*
	21164	Blood	64	0.25		34	*blaZ*
	21165	Blood	127	2	Rifampin, fusidic acid	34	*blaZ*
	21166	Blood	131	0.25	Rifampin, fusidic acid	34	*blaZ*
	23506	Blood	263	0.5		34	*blaZ*

### Comparative genomics of clinical isolates.

To investigate the mechanism of treatment failure and secondary increase in oxacillin MIC, we sequenced 7 and 6 isolates from cases 1 and 2, respectively. We also generated a complete, fully assembled reference genome of the index isolate of case 1 (AUS0325), since a close reference was not available in public databases (17). The *in silico* resistome confirmed that neither isolate had acquired the *mecA* or *mecC* elements; however, all were positive for the β-lactamase gene (*blaZ*). The *in silico* multilocus sequence type (MLST) profile showed that isolates from the same patients belonged to the same ST (case 1, ST88; case 2, ST34). Moreover, the ST-specific phylogeny of the clinical isolates supplemented with Australian and international S. aureus genome sequences showed that the clinical isolates belonged to a *blaZ*-positive MSSA lineage and that same-patient isolates were monophyletic (see Fig. S1 in the supplemental material). Thus, in both cases, sequencing data were consistent with a persistent infection rather than reinfection with a new MSSA clone.

10.1128/mBio.02882-20.2 FIG S1 ST-specific phylogeny showing close relatedness of clinical isolates in case 1 (ST88) and case 2 (ST34). Download FIG S1, PDF file, 0.2 MB. Copyright © 2020 Giulieri et al. 2020 Giulieri et al. This content is distributed under the terms of the Creative Commons Attribution 4.0 International license.

**FIG 2:**
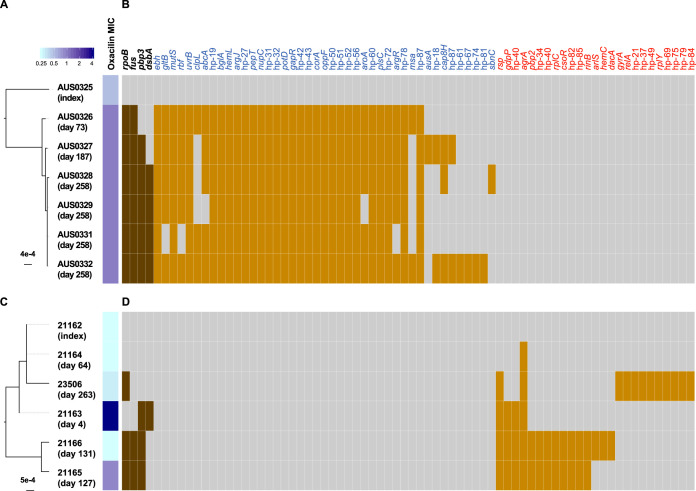
Comparative genomics of clinical isolates (case 1 in panels A and B and case 2 in panels C and D). Maximum likelihood phylogenies (A and C) specific to the clinical episodes were inferred from a curated alignment after excluding SNPs based on coverage and fraction of the alternate allele (see Materials and Methods for details). After excluding intergenic and synonymous mutations, 48 and 36 mutations altering protein sequences were identified in cases 1 and 2, respectively (B and D). While 68 genes were mutated only in case 1 or 2, four genes were convergently mutated in both cases.

Using an episode-specific variant calling strategy to identify mutations separating subsequent isolates from the index isolate (i.e., collected at start of the infection), we identified 95 mutations (across 6 isolates) and 52 mutations (across 5 isolates) in cases 1 and 2, respectively. Of these, 48 mutations and 36 mutations were predicted to alter protein sequences in cases 1 and 2, respectively (Fig. 2). A complete list of mutations is provided in Table S2 in the supplemental material.

As we have previously shown that structural variants can frequently occur in S. aureus, notably over the course of persistent S. aureus infection (18–20), we analyzed the pangenome as well as read coverage to detect larger chromosomal variants. Analysis of case 2 isolates revealed two clusters of contiguous genes, including a total of 45 genes that were absent from isolates 21165 and 21166, collected more than 100 days after the index isolate (Fig. 1). Read coverage inspection using reference Sa_ILRI (ST30) (21) and whole-genome alignment confirmed a large 30-kb deletion corresponding to the prophage Sa ϕ2, indicating phage excision occurring late over the course of the infection. All deleted genes were hypothetical proteins or encoded phage proteins.

Taken together, comparative genomics revealed that a high number of genetic changes arose during the prolonged infections and multiple antibiotic treatments, pointing to adaptive evolution of the clone within the host under the pressure of antibiotic treatment and the immune system (22).

### Convergence among mutations in clinical isolates.

To identify genes under convergent evolution as a signature of bacterial adaptation during persistent infections, we clustered mutated genes and found that four genes were mutated in both clinical cases, albeit with different mutations (Fig. 2). Two of these genes were the known resistance loci *fusA* and *rpoB*, which are associated with fusidic acid resistance and rifampin resistance, respectively. The *fusA*-M401T mutation in case 1 has not been previously described in clinical isolates; however, the domain encompassed by positions 404 to 483 is a hot spot for fusidic acid resistance mutations *in vitro* (23). Mutations have been also found *in vitro* at positions 1 to 280, as in one isolate in case 2 (23). Mutations of the *rpoB* gene at positions 477 and 527 in case 1 and 471 in case 2 have been previously described in rifampin-resistant S. aureus isolates or mutants (24).

Mutations in the penicillin-binding protein 3 gene (*pbp3*) were also detected in isolates from both cases. Point mutations in penicillin-binding proteins (especially *pbp2*) are thought to be associated with oxacillin resistance (10, 25). The fourth convergent gene (*dsbA*) codes for a thiol disulfide oxidoreductase. This enzyme modifies peptides posttranslationally by generating disulfide bonds (26).

Among the nonconvergently mutated loci, it was worth noting an N344S mismatch DNA repair gene *mutS* mutation in case 1. While this locus is not linked with antibiotic resistance *per se*, the mutation may have promoted a hypermutator phenotype (27) and might explain the high number of mutations compared to other within-host studies (19). Interestingly, clinical isolates in case 2 had mutations in *pbp2* (G134D substitution) and *gdpP* (Q572 frameshift), both previously linked to oxacillin resistance (10, 15).

### MSSA adaptations to oxacillin arise at high frequency *in vitro* but have strong impacts on fitness.

**FIG 3:**
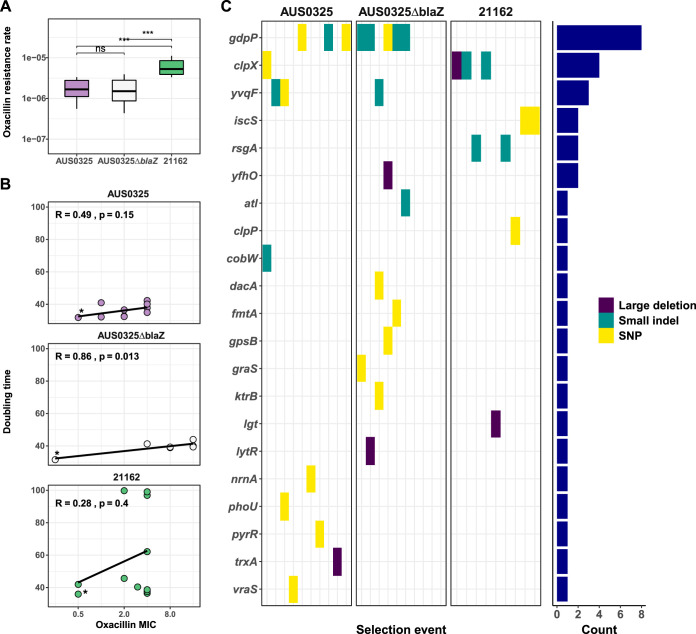
Outcome of 30 independent oxacillin adaption *in vitro* experiments. (A) Rate of oxacillin resistance selection across three different backgrounds (AUS0325, case 1 index isolate; AUS0325Δ*blaZ*: case 1 index isolate with Δ*blaZ* mutation; 21162, case 2 index isolate). Ten selection experiments were performed on each background. Comparison of rates of resistance induction between the 3 backgrounds was done by Mann-Whitney test. (B) Scatterplot showing the correlation between oxacillin MIC (in mg/liter) and doubling time in heart infusion (HI) broth. The wild-type isolate is represented by an asterisk. (C) Heat map of 21 protein genes that were mutated after *in vitro* selection. Mutations are colored according to the type (single-nucleotide polymorphisms [SNP], small indels [below 20 bp], and large deletions).

Since several loci could potentially be linked to oxacillin adaptation in the clinical isolate cases, we used experimental evolution to generate mutants associated with reduced oxacillin susceptibility. Ten independent resistance selection experiments were performed at an oxacillin concentration of 2 mg/liter in three genetic backgrounds (case 1 index isolate, case 2 index isolate, and case 1 index isolate with *ΔblaZ* mutation), with the aim of obtaining 30 independently selected oxacillin-resistant mutants. After two passages on oxacillin agar plates, we generated 26 independent mutants with an oxacillin MIC up to 16 g/liter. Four clones had a MIC of <2 g/liter (Fig. 3). Selection experiments in a β-lactamase-negative background were more challenging: in four cases we were unable to obtain sufficient colonies for sequencing and susceptibility testing, and in the remaining case, generation of mutants required a third passage, resulting in mutants with a higher oxacillin MIC than in the β-lactamase-positive backgrounds (medians of 4 and 12 mg/liter, respectively; *P* = 0.007, Kruskal-Wallis test). While *in vitro*-adapted mutants remained susceptible to vancomycin and daptomycin, we observed an increase of the cefazolin MIC in mutants selected in the β-lactamase-negative background, suggesting that a higher oxacillin MIC (≥4 mg/liter) can be associated with cross-resistance to cephalosporins (see Table S1 in the supplemental material).

Oxacillin resistance was easily selected with single-step exposure experiments. We found that the mutant frequency was around 10^−6^ (median of 10 experiments: AUS0325 background, 1.9 × 10^−6^ and 1.5 × 10^−6^; 21162 background, 5.2 × 10^−6^). This is similar to the mutation rate to antibiotics such as rifampin and ciprofloxacin; however, this resistance mechanism selected by oxacillin exposure had a strong fitness impact. The growth of oxacillin-adapted mutants in heart infusion (HI) broth was significantly impaired, with median increases of doubling times of 5.3, 8.2, and 7.3 min compared to the wild type (Fig. 3A). Furthermore, there was a correlation between oxacillin MIC and doubling time, although this was statistically significant only for one of the three backgrounds tested. Similarly, a similar growth impairment was also observed in adapted strains from the clinical cases 1 and 2 (median increases in doubling times of 6.7 and 32.5 min, respectively) (see Table S1 in the supplemental material).

10.1128/mBio.02882-20.7 TABLE S1 List of strains included in this study (parts 1 and 2) and list of strains used for ST88 and ST34-specific phylogenies (part 3). Download Table S1, XLSX file, 0.04 MB. Copyright © 2020 Giulieri et al. 2020 Giulieri et al. This content is distributed under the terms of the Creative Commons Attribution 4.0 International license.

10.1128/mBio.02882-20.8 TABLE S2 List of mutations in isolates from clinicals cases with secondary increase in oxacillin MIC and in *in vitro* adapted oxacillin-resistant mutants. Download Table S2, XLSX file, 0.03 MB. Copyright © 2020 Giulieri et al. 2020 Giulieri et al. This content is distributed under the terms of the Creative Commons Attribution 4.0 International license.

### Convergence analysis reveals a strong signature of adaptation to oxacillin.

Genome sequencing of the 26 *in vitro*-adapted isolates revealed 34 small mutations (single nucleotide polymorphisms [SNPs] or small indels) and five large deletions ranging from 57 to 423 bp (Table S2). Sixteen isolates had one mutation, eight isolates had two mutations, one isolate had three mutations, and one isolate had four mutations (see Fig. S2 in the supplemental material). Mutants selected in the β-lactamase-negative background had a higher number of mutations (median of 2 versus 1; *P* = 0.0002), probably as a consequence of the additional passage on oxacillin plate. Three point mutations were found in two independently selected mutants, indicating convergence at the mutation level. Among 31 unique point mutations, 28 (91%) were predicted to alter protein sequences, through either amino acid substitutions (16 mutations), truncation (one mutation), or frameshift mutations (11 mutations), suggesting positive selection.

10.1128/mBio.02882-20.3 FIG S2 Network of *in vitro* mutants, selected on oxacillin at 2 mg/liter and on three genetic backgrounds. Nodes are colored according to oxacillin MIC (deep blue, highest MIC; light blue, lowest MIC). Small nodes represent hypothetical intermediate genotypes. Edges are colored according to the type of mutation (light blue, synonymous substitutions; red, missense substitutions; black, frameshift/nonsense mutations; gray, mutations in noncoding regions). Download FIG S2, PDF file, 0.1 MB. Copyright © 2020 Giulieri et al. 2020 Giulieri et al. This content is distributed under the terms of the Creative Commons Attribution 4.0 International license.

Among the 21 genes that were mutated after oxacillin selection, two had the highest signature of convergent evolution. The c-di-AMP phosphodiesterase gene *gdpP*, whose role in oxacillin resistance has been recently explored (15), was mutated in 8 out of 26 independently selected mutants and in the two different genetic backgrounds. The ATP-dependent protease genes *clpXP*, encoding a molecular chaperone, also previously linked to oxacillin resistance (28), were also mutated in multiple experiments (*clpX* in 4 isolates and *clpP* one isolate). Further genes selected more than once included *yvqF/vraT*, a predicted membrane protein gene located upstream of *vraRS* (3 isolates) (29), the cysteine desulfurase gene *iscS*, and the ribosome biogenesis GTPase gene *rsgA* (both mutated in 2 isolates).

This selection experiment has revealed a network of genes selected for by the oxacillin treatment and tightly linked by direct or indirect involvement in the cell wall metabolism. This not only applies to *gdpP* and *clpXP*, but also to the two-component regulatory systems *vraRS* and *graRS*, which are known determinants of vancomycin resistance in S. aureus (30, 31).

Importantly, no protein-altering or intergenic mutations affecting the *blaZ* operon or PBPs were identified. Thus, this *in vitro* evolution experiment didn’t provide evidence for the two classic paradigms of low-level oxacillin resistance as the primary mechanism. This is further underscored by the selection in the β-lactamase-negative background, which indicates that oxacillin resistance could be selected at the same rate as in the β-lactamase-positive strains and that there were at least 10 genetic loci promoting oxacillin adaptation, some of them coinciding with genes on selective pressure in β-lactamase-positive backgrounds (Fig. 3C).

### Distribution of S. aureus oxacillin MICs across a phylogeny of 490 *mec*-negative S. aureus isolates suggests recurrent but nonpersistent adaptation to β-lactams.

**FIG 4:**
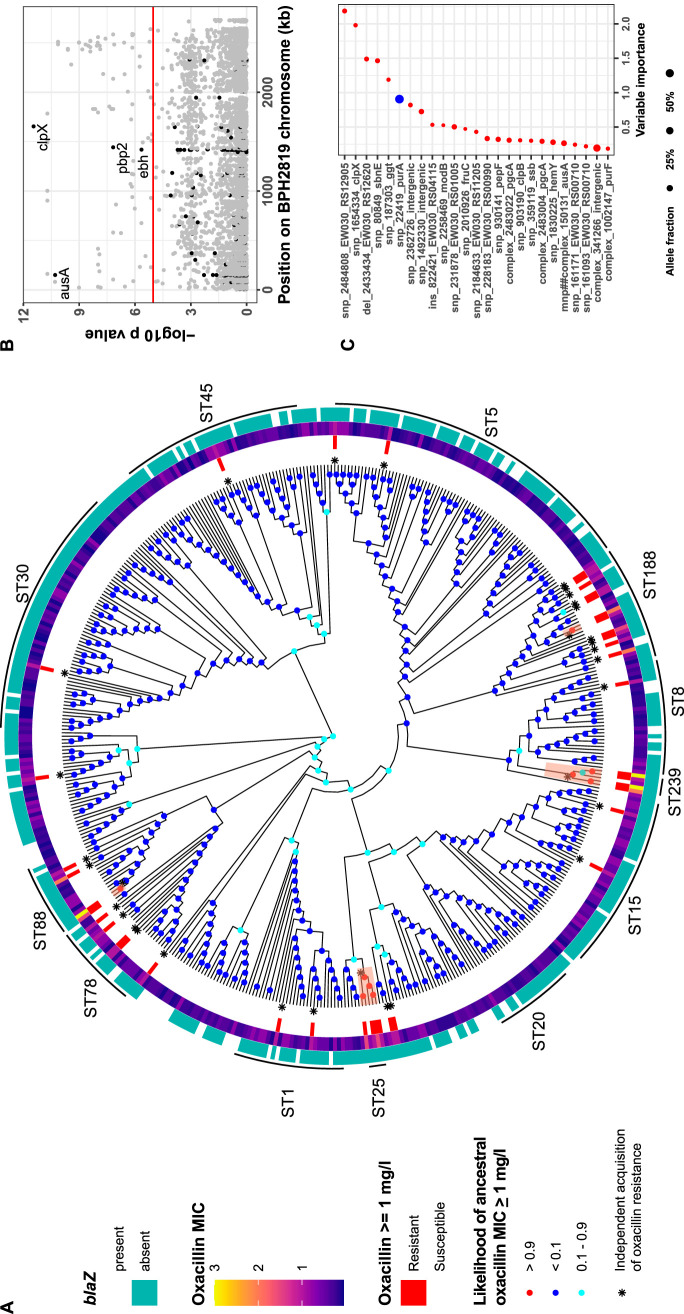
Oxacillin resistance (≥1 mg/liter) in a collection of methicillin-susceptible (*mec* negative) S. aureus strains. (A) Maximum likelihood cladogram inferred from the core genome alignment. Internal nodes are colored according to likelihood of the ancestral state of oxacillin resistance. Independent acquisitions of oxacillin resistance (internal nodes or terminal nodes) are depicted by an asterisk; oxacillin resistance, the oxacillin MIC, and the presence/absence of *blaZ* are shown on the internal, middle, and outer rings, respectively. (B) Manhattan plot of 5,276 variants, displayed by position on the reference genome and significance of the association with high oxacillin MIC (univariate analysis using a linear mixed model). Variants in genes mutated in clinical cases and in *in vitro*-selected strains are colored in black. The red line shows the Bonferroni-corrected significance threshold. Labeled genes reached the Bonferroni significance threshold and were mutated in clinical cases or in *in vitro*-selected strains. (C) Top 25 mutations ranked by highest importance based on the impurity index (red circles or blue circles indicate mutations associated with an increase or decrease of oxacillin MIC, respectively). Mutation labels were constructed by concatenating the type of mutation, position on reference BPH2819, and gene name (or BPH2819 locus tag).

To broaden the exploration of low-level oxacillin adaptation, we investigated a collection of 490 previously described *mec*-negative S. aureus clinical isolates obtained from two cohort studies of S. aureus bacteremia (4, 32) and a genomic study of multidrug-resistant microorganisms (33). The majority of the isolates had a low MIC (median, 0.38 mg/liter; interquartile range [IQR], 0.19 to 0.38 mg/liter); however, 40 of them (∼8%) had reduced oxacillin susceptibility (MIC of ≥1 mg/liter) (Fig. 4A; see Fig. S3 in the supplemental material). Mapping of the oxacillin MIC on the maximum likelihood phylogenetic tree showed that the high MIC occurred sporadically along the branches, suggesting recurrent adaptation to oxacillin in the MSSA population. Previous reports have described clones of S. aureus with a higher oxacillin MIC, such as ST25 (10). Here, resistant isolates appeared to be more prevalent in some lineages (e.g., ST188, ST239, and ST25); however, using ancestral state reconstruction of high oxacillin MICs, we found only 9 internal nodes with a high inferred likelihood of resistance. Overall, this supports multiple recurrent acquisitions of the phenotype (30 independent acquisitions among 40 resistant isolates), with four small monophyletic clades (two to five isolates), generated by a resistant ancestor, suggesting that limited transmission might have been possible.

10.1128/mBio.02882-20.4 FIG S3 Distribution of oxacillin MICs (mg/liter) across 490 clinical isolates. Red bars represent concentration at or above 1 mg/liter, the cutoff that was chosen to separate low from high oxacillin MICs. Download FIG S3, PDF file, 0.1 MB. Copyright © 2020 Giulieri et al. 2020 Giulieri et al. This content is distributed under the terms of the Creative Commons Attribution 4.0 International license.

Thus, despite the enrichment within specific sequence types, the phylogeny does not support the presence of large stable, adapted low-level-resistant lineages. Furthermore, the pattern of low-level oxacillin resistance and adaptation within a diverse MSSA population recapitulates our observations in the *in vitro*-adapted mutants. The high mutation rate is reflected in the recurrent acquisition of the phenotype, while the high fitness cost likely prevents the constitution of stable and transmissible adapted lineages. An analogous resistance instability could be observed within the host in clinical case 2, where oxacillin resistance reverted in the absence of exposure to flucloxacillin (Fig. 1B).

### The univariate GWAS identifies and flags potential targets associated with oxacillin adaptation.

To identify recurring mutations associated with increased oxacillin MIC, we applied a genome-wide association study (GWAS) approach to the collection of 490 MSSA isolates (Fig. 4B). To account for the highly clonal population structure of the collection, we first removed nonhomoplastic variants from the list of core genome mutations to reduce the data set to 5,276 variants that were acquired at least twice across the phylogeny. We then applied a linear mixed model using a kinship matrix as a random effect. After correcting for multiple testing, the analysis identified 95 mutations (64 genes) with a significant positive (93 genes) or negative (2 genes) association with oxacillin resistance (as a binary variable with a breakpoint at 1 mg/liter).

A limitation of GWAS is the risk of identifying significantly associated mutations that are not causative of the phenotype. Also, a false-positive association can arise due to residual population structure (34). We reasoned that true-positive variants could be identified by intersecting the GWAS results with our other genomic approaches. Interestingly, we found one mutation in a gene that was recurrently mutated in *in vitro*-adapted mutants (*clpX*-P101S) and in three genes that were flagged both in the GWAS and in the clinical cases, including the *pbp2*-G629P mutation (see Table S3 in the supplemental material). The *clpX*-P101S mutation was found in eight isolates belonging to two different clonal complexes (indicating convergent evolution) and led to a median oxacillin increase from 0.38 to 1 mg/liter. Together with the strong convergence *in vitro* (the gene with the second strongest signature of convergence with four independent selection events, including a large deletion) and evidence from the literature (28), this suggests a role of *clpX* in low-level oxacillin resistance, notwithstanding the absence of mutations in the two clinical cases. Overall, these results show that some adaptive responses (like *clpX* mutations) identified from within-host and *in vitro* evolution studies can be confirmed by population-level comparative genomics, while others (such as *gdpP* mutations) are less apparent—possibly due to the fitness effects on the stability of those substitutions.

10.1128/mBio.02882-20.9 TABLE S3 Top 91 mutations from the univariate GWAS analysis of high oxacillin MIC, selected based on a Bonferroni corrected *P* value of <0.05. Download Table S3, XLSX file, 0.02 MB. Copyright © 2020 Giulieri et al. 2020 Giulieri et al. This content is distributed under the terms of the Creative Commons Attribution 4.0 International license.

To assess the predictive ability of the top 95 genetic signatures identified in the GWAS analysis, we trained a classification model of high oxacillin MIC using the random forest (RF) algorithm. The model was able to predict oxacillin resistance in the test data set with an area under the curve of 0.81 (sensitivity, 66%; specificity, 89%) (see Fig. S4 in the supplemental material), supporting the relevance of the GWAS findings (high specificity); however, it also indicates that the mutations identified cannot predict the phenotype in about one-third of the resistant isolates.

10.1128/mBio.02882-20.5 FIG S4 Receiver operator characteristic (ROC) curves of the random forest model trained on 95 mutations that were above the Bonferroni-corrected significance threshold in the GWAS analysis. The performances of the model on the train and test data sets are shown in blue and red, respectively. Download FIG S4, PDF file, 0.1 MB. Copyright © 2020 Giulieri et al. 2020 Giulieri et al. This content is distributed under the terms of the Creative Commons Attribution 4.0 International license.

While this first model needs to be tested with a new and totally independent data set to assess its true ability to predict MIONSA, the variable importance ranking of RF allows us to identify predictors that have a stronger link with MIONSA in our data set (35).

The most important variant was an M15V mutation in a hypothetical protein (RS12905). Interestingly, this approach confirmed the association of *clpX*-P101S with high oxacillin MIC (the second most important feature in the model [Fig. 4]), while other important features were an in-frame deletion in a NAD-dependent oxidoreductase (RS12620) and a P395S mutation in the gene *ggt* coding for γ-glutamyltransferase, a metabolic enzyme that can transfer γ-glutamyl groups to amino acid residues (transpeptidation) (36) and which has been linked to capsule anchoring to peptidoglycan in Bacillus anthracis (37). Of note, a mutation in *clpB* had also a high importance ranking. This gene is part of the same family of Clp ATPases to which *clpX* belongs and plays an essential role in stress tolerance (e.g., heat shock) (38).

### Experimental validation of key mutated targets responsible for oxacillin adaptation.

**FIG 5:**
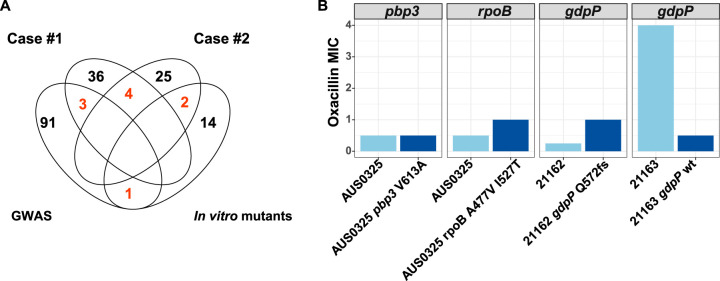
Overlap among genomic approaches and experimental validation of top genes associated with oxacillin adaptation. (A) A total of 10 genes were found to be recurrently linked to oxacillin adaptation using different genomic approaches. Four genes (*pbp3*, *rpoB*, *fusA*, and *dsbA*) were mutated in both clinical cases, while the two genes *gdpP* and *dacA* were altered in one clinical case and in the *in vitro*-adapted strains. The univariate GWAS of 490 clinical strains identified one gene (*clpX*) that was also under oxacillin pressure *in vitro* and 3 that were mutated in clinical isolates. (B) Selected mutations in genes that were recurrently mutated were investigated using site-directed mutagenesis. Mutations detected in clinical cases (*pbp3*-V613A, *rpoB*-A477V 1527T, and *gdpP*-Q572fs) were reconstructed using a clinical isolate as the background (light blue, wild type; dark blue, mutant).

Our approach allowed us to detect genes associated with oxacillin adaptation *in vitro*, in clinical cases of oxacillin failure, and among a diverse collection of bacteremia isolates (Fig. 5A). We next used targeted mutagenesis to assess the role of these genes in oxacillin and reasoned that genes that were recurrently mutated in clinical cases or *in vitro* would be the best targets of oxacillin adaptation. First, we reconstructed the *pbp3*-V613A mutation in the AUS0325 background (case 1, index isolate), but there was no change in the oxacillin MIC compared to AUS0325, indicating that the *pbp3* mutation was not responsible for the increase in oxacillin MIC. As *rpoB* mutations have also been associated with reduced susceptibility to oxacillin (24, 39, 40), we also reconstructed the *rpoB* allele A477V I527T using the same background: the oxacillin MIC increased slightly from 0.5 to 1 mg/liter by broth microdilution and from 0.75 to 1 mg/liter by Etest. Overall, there was either no change or very limited increase in oxacillin MIC in *pbp3* and *rpoB* mutants.

When considering genes identified through *in vitro* selection, we found that the *gdpP* gene that was recurrently mutated in the *in vitro*-adapted isolates was also mutated in 3 isolates of case 2. One of these isolates (21163, collected on day 4) had an oxacillin MIC of 4 mg/liter and was genetically closely related to the index isolate (oxacillin MIC of 0.25 mg/liter), suggesting that the *gdpP* deletion at position 572 was responsible for the increased oxacillin resistance. To confirm this hypothesis, we used allelic exchange to insert the wild-type *gdpP* allele into 21163. Compared to 21163, the isogenic mutant 21163 with the *gdpP* wild type had an oxacillin MIC of 0.5 mg/liter (Fig. 5B).

Thus, *gdpP* was a key gene in acquisition of oxacillin resistance in case 2. When combining mutations identified *in vitro* and in clinical isolates, we observed that the mutations were concentrated in two specific regions (see Fig. S5 in the supplemental material). The first region was the GGDEF domain of *gdpP*, and the second included the domain DHHA1 and a 100-amino-acid (aa)-long sequence immediately upstream of it. Through its phosphodiesterase activity, *gdpP* degrades the second messenger c-di-AMP, which is generated by the diadenyl-cyclase gene *dacA* (76). Interestingly, *dacA* was the second gene that was mutated in both clinical isolates and *in vitro* mutants (mutations T39S and V78G, respectively). In clinical case 2 and in the *in vitro*-adapted mutants, *dacA* mutations were not the only mutations separating the isolates from the wild type (or index isolate); thus, it is unclear whether *dacA* mutations contribute to oxacillin resistance or rather are compensatory.

10.1128/mBio.02882-20.6 FIG S5 (A) Alignment of 9 unique mutated *gdpP* alleles in 11 isolates (3 clinical isolates from case 2 and 8 *in vitro* mutants). Point mutations are depicted as black vertical (substitutions and insertions) or horizontal (deletions) traits. Premature stop codons are indicated by an asterisk. The index isolate for each case 2 isolate and the *in vitro* selection is written in oblique characters. Clinical isolates 21163, 21165, and 21166 and *in vitro* mutants BPH3824, BPH3830, BPH3832, and BPH3833 have supplementary mutations separating them from the index isolate. (B) Summary of *gdpP* mutations identified in case 2 and in the *in vitro* mutants mapped on the protein domain architecture. TM, transmembrane domain; PAS, PAS sensory domain; GGDEF, GGDEF domain (thought to have regulatory function); DHH, DHH domain (phosphodiesterase activity). Download FIG S5, PDF file, 0.2 MB. Copyright © 2020 Giulieri et al. 2020 Giulieri et al. This content is distributed under the terms of the Creative Commons Attribution 4.0 International license.

## DISCUSSION

Despite the frequency of MSSA infections, little is known about bacterial factors associated with treatment outcome with antistaphylococcal penicillins, and the impact of subtle changes in oxacillin MIC has not been studied extensively, unlike vancomycin for MRSA infections. Our clinical cases indicate that oxacillin failure might be associated with secondary increase in oxacillin resistance that is unrelated to the presence of the *mec* gene.

Under the selective pressure of oxacillin, MSSA isolates undergo a phenotypic adaptation that is characterized not only by decreased susceptibility but also by slower growth. This adaptive phenotype appears to arise frequently but is also generally unstable, as indicated by the reversions to the wild type in clinical cases and the absence of large, stable oxacillin-resistant lineages, although transmission of low-level oxacillin-resistant strains has been reported, for example, in a dermatological ward (41). This is in contrast with other forms of mutational resistance such as rifampin resistance, where large S. aureus clades carrying a single *rpoB* mutation appeared to have spread globally (24). Thus, these strains share similarities with vancomycin-intermediate S. aureus (VISA). Similarly to VISA, the oxacillin-adapted *mec*-negative strains seem to be preferentially selected in chronic, deep-seated MSSA infections with incomplete source control. This suggests the need for a prolonged exposure of a high bacterial load to oxacillin and is consistent with the fitness cost of oxacillin adaptation. At least two previously published reports illustrate persistent infections similar to the clinical cases investigated here (42, 43). To take into account both low-level resistance and the lack of the *mec* gene, we suggest labeling these strains with the acronym MIONSA (*mec*-independent oxacillin-nonsusceptible S. aureus). While MIONSA can be broadly described by low-level oxacillin resistance in MSSA (i.e., in the absence of the *mec* gene or phenotypic features associated with it), there are no defined breakpoints to separate susceptible strains from oxacillin-intermediate strains (7). The clinical implications of the phenotype are not fully determined (44, 45); however, the association with flucloxacillin failure identified by us and others (42, 43) suggests that it is preferable to avoid treating serious infections with flucloxacillin. Since our data indicate a risk of coresistance to cefazolin but not to vancomycin or daptomycin, it is probably better to avoid β-lactams in this setting.

The low-level oxacillin resistance phenotype has been recognized for decades (9) and related mechanistically to either β-lactamase hyperproduction (BORSA) or PBP mutations (MODSA) (8, 14). Tomasz et al. showed that some clinical strains with oxacillin MICs of 1 to 4 mg/liter, lacking PBP2a, and with a homogenous resistance profile had penicillin-binding proteins with reduced affinity to penicillin (14). It was later shown that one of those strains carried point mutations near the penicillin-binding motif of *pbp2* (46). Other studies have shown that point mutations in penicillin-binding protein genes might be associated with increased resistance to oxacillin and other β-lactamase-resistant penicillins (10, 25). In one study of 38 strains with oxacillin MICs of 1 to 8 mg/liter and different genetic backgrounds, the transpeptidase domain of *pbp2* was polymorphic. Transformation of a fully susceptible strain with *pbp2* from a resistant strain increased the oxacillin MIC from 0.25 to 4 mg/liter; since no whole-genome sequencing was performed, it is not clear whether mutations in other genes were acquired during mutagenesis (10). Using whole-genome sequencing, Ba et al. investigated 4 *mecA*-negative S. aureus isolates with oxacillin MICs of 2 to 4 μg/ml and identified point mutations in *pbp1*, *pbp2*, and *pbp3*; however, the impact of these mutations was not assessed by mutagenesis (25).

Our results, strengthened by a comprehensive genomic approach and targeted mutagenesis, challenge both classic paradigms of low-level oxacillin resistance. With regard to the β-lactamase hypothesis, we showed that oxacillin resistance is relatively easily selected in a β-lactamase-negative mutant. Furthermore, no adaptative mutations were identified in the *bla* operon or its promoter region. We discovered, however, that the β-lactamase-negative oxacillin-adapted strains were more unstable and that in a large collection of bacteremia isolates, the high oxacillin MIC phenotype was found only in those that carried the *blaZ* gene. Taken together, these findings indicate that while it is not the primary driver of the phenotype, the β-lactamase might facilitate or stabilize oxacillin resistance. As for PBP mutations, we were initially struck by the presence of *pbp3* mutations in both clinical cases. However, we constructed a mutant carrying the *pbp3* mutation identified in resistant isolates of case 1 and could not reproduce the increase in oxacillin MIC. The lack of association with PBP mutations is supported by the fact that these genes were not mutated in *in vitro*-adapted mutants. Nevertheless, the findings in the 2 clinical cases and the GWAS and data from previous publications suggest that PBP genes might be under selective pressure in isolates with a high oxacillin MIC. It is possible that these mutations may have a compensatory role.

The genetic mechanism of MIONSA appears to be polygenic. Here, the generation and sequencing of 26 independently selected *in vitro* oxacillin-adapted mutants suggested that it is linked to mutations in genes involved in c-di-AMP signal transduction pathways (*gdpP* and *dacA*), in the *clpXP* chaperone-protease complex, or in regulators such as the two-component systems *vraRS* and *graRS*.

These findings are consistent with two recent studies that have identified a wide range of *gdpP* mutations (in particular gene truncations) in MIONSA isolates obtained from both human and animal samples (15, 16). This is further underscored by *in vitro* selection studies that have identified *gdpP* mutations upon exposure to oxacillin in MRSA (47, 48) and MSSA (49–51) backgrounds. Furthermore, the involvement of *gdpP* is not limited to oxacillin; importantly, *gdpP* mutations have also been linked to ceftaroline resistance (52). The function of *gdpP* is to hydrolyze the signal nucleotide c-di-AMP, which was shown to be involved in cell wall homeostasis. Corrigan et al. discovered that *gdpP* truncations allowed lipoteichoic acid-deficient MRSA mutants to grow normally (suppressor strains) (53). These mutants exhibited increased MICs for lysostaphin, oxacillin, and penicillin, further highlighting the link between *gdpP* activity and cell wall metabolism. In our study, we found a discrepancy of our genomic approaches: while *gdpP* truncations were prominent in clinical cases and *in vivo* mutants, no truncation was found in 490 clinical strains, and mutations were not associated with high oxacillin MICs in the GWAS. The most likely explanation is that *gdpP* truncations carry a high fitness cost (as demonstrated by our data) and that they quickly revert to the wild type in the absence of oxacillin pressure.

An important finding of this study is the key role of *clpX* mutations in providing low-level oxacillin resistance. This gene was mutated in four independent selection experiments and ranked very high both in the GWAS after adjustment for population structure and in the random forest model of high oxacillin MICs in a collection of 490 clinical *mec*-negative isolates. ClpX is a bacterial molecular chaperone that can associate with ClpP to act as a protease (54). Interestingly, recent work has shown that mutations inactivating the *clpXP* complex in the USA300 MRSA strain lead to increased oxacillin resistance, thickened cell wall, and enhanced cross-linking of peptidoglycan (28). Mutagenesis experiments also indicated a role of *clpX* in regulation of autolysis, cell division (42), and virulence in skin infections (55, 56).

By combining sequencing of clinical isolates, *in vitro* evolution experiments, large-scale analysis of a broad population of MSSA strains, and targeted mutagenesis to confirm key mutations, we were able to provide a comprehensive description of an important adaptive phenotype that might be linked to treatment failure in severe, deep-seated MSSA infections. We challenge the classic paradigm of MIONSA and establish a list of core genes (including those involved in the c-di-AMP pathway, the *clpXP* complex, and other loci linked to cell membrane metabolism) (Table S2) associated with oxacillin adaptation *in vitro*, which can be used to establish genotype-phenotype correlations. Since it is likely that other pathways can lead to the MIONSA phenotype, it will be important to set up a prospective surveillance of clinical non-*mec*-mediated low-level oxacillin resistance. Finally, clinicians and microbiologists should be aware of this important phenotype in persistent or recurrent severe MSSA infections. While currently MIONSA is more likely to be identified through culture-related techniques, in the future, rapid bacterial whole-genome sequencing might allow early identification of high-risk mutations and for treatment to be targeted accordingly.

## MATERIALS AND METHODS

### Strains and growth conditions.

Forty-eight S. aureus strains included in this study (listed in Table S1) were stored in glycerol broth at −80°C and subcultured onto horse blood agar (HBA) before use. All isolates were grown in heart infusion (HI) broth.

### Antibiotic susceptibility testing.

The oxacillin MIC was determined using the Etest (bioMérieux) according to the manufacturer’s instructions and using the broth microdilution (BMD) method as described in CLSI guidelines (57). For BMD, strains were grown in cation-adjusted Mueller-Hinton broth supplemented with 2% NaCl and oxacillin at the following concentrations: 0.06, 0.12, 0.25, 0.5, 1, 2, 4, 8, 16, and 32 mg/liter. S. aureus ATCC 29213 was used as the quality control. Testing was performed in duplicates and repeated a third time in case of disagreement. Vancomycin and daptomycin MICs were determined using the Sensititre system (GPN3F panel; Trek Diagnostic Systems, Thermo Fisher Scientific) as per the manufacturer’s instructions. Colony material from an overnight culture was emulsified in sterile water, and the suspension was adjusted to a 0.5 McFarland standard using the Vitek DensiChek densitometer (bioMérieux). Thirty microliters of the 0.5 McFarland suspension was transferred into 11 ml cation-adjusted Mueller-Hinton broth provided by the manufacturer, of which 50 μl was inoculated into each well of the plate. The plates were incubated for 20 to 24 h at 35°C, and results were read using the Sensititre Vizion Digital MIC viewing system (Trek Diagnostic Systems, Thermo Fisher Scientific). The GPN3F panel has a vancomycin range from 1 to 128 mg/liter and a daptomycin range from 0.25 to 8 mg/liter. The cefazolin MIC was determined using the agar dilution method, following CLSI guidelines (57). Cefazolin was tested in serial 2-fold dilutions ranging from 0.25 to 16 mg/liter on Mueller-Hinton agar. The plates were inoculated with a 1-mm-pin multipoint applicator that delivered approximately 1.0 × 10^4^ CFU per spot and then incubated at 35°C in ambient air for 20 to 24 h. The MICs were determined as the lowest concentration of drugs that completely inhibited colony formation. The following organisms were used for quality control: Escherichia coli ATCC 25922 (cefazolin), Staphylococcus aureus ATCC 29213 (cefazolin, daptomycin, oxacillin, and vancomycin), and Enterococcus faecalis ATCC 29212 (daptomycin and vancomycin).

### Growth rate analysis.

Isolates grown overnight were inoculated into 200 μl of HI broth at a 1:400 dilution and incubated at 37°C with agitation during 16 h. Optical density at 600 nm was measured at 15-min intervals using the EnSight Multimode plate reader (PerkinElmer). Doubling time and maximum grow rate were calculated by fitting curves using local regression as performed by the R package cellGrowth (59).

### *In vitro* selection of oxacillin-adapted mutants.

Ten single colonies from HBA plates of the AUS0325, 21162, and AUS0325 Δ*blaZ* isolates were used to inoculate 10 independent overnight cultures in HI broth. For each culture, inoculums of 100 μl at 10^7^ and 10^8^ CFU/ml (confirmed by colony count) were spread onto Mueller-Hinton agar supplemented with 2% NaCl and 2 mg/liter oxacillin. Colonies on antibiotic selective plates were counted, and one single colony per experiment was subcultured onto Mueller-Hinton agar supplemented with 2% NaCl and 2 mg/liter oxacillin. Ten to 20 colonies were resuspended in 1.5 ml phosphate-buffered saline. Half of the suspension was stored in glycerol at –80°C, and half was centrifuged at 8,000 × *g* for 5 min, and the pellet was stored at –20°C until DNA extraction. The selection experiment was performed on three different backgrounds (AUS0325, the index isolate of clinical case 1; 21162, the index isolate of case 2; and AUS0325 Δ*blaZ*), resulting in 30 independent selection events.

### Whole-genome sequencing.

Genomic DNA was extracted from single colonies (clinical isolates) or bacterial suspensions as described above (*in vitro*-selected mutants) using the Janus automated workstation (PerkinElmer) or manually using the DNeasy blood and tissue kit (Qiagen). The DNA concentration was normalized to 0.2 ng/μl for library preparation with Nextera XT DNA (Illumina). Genome sequencing was performed on MiSeq and NextSeq (Illumina). To assess the quality of the sequences, mean read depth, assembly metrics using Spades v3.7.1 (58), and the percentage of S. aureus reads using Kraken v0.10.5 beta (60), were calculated.

### Multilocus sequence typing and resistome.

We used MLST v2.6 (T. Seemann [https://github.com/tseemann/mlst]) to determine the multilocus sequence type (MLST) *in silico* from *de novo* assemblies of the isolates (obtained using Spades). Resistance genes were identified in assemblies using Abricate v0.3 (T. Seemann [https://github.com/tseemann/abricate]) and the ResFinder database (61), with a coverage and identity threshold of 90%.

### MLST-specific phylogenies.

To construct phylogenetic trees specific to the sequence types of cases 1 and 2, reads were mapped to the PacBio reference Sa_AUS0325 (case 1, ST88) and Sa_FORC_001 (case 2, ST34) using the Snippy pipeline v4.4.3 (T. Seemann [https://github.com/tseemann/snippy]). The core genome alignment obtained with snippy-core was used to infer the phylogeny with IQ-TREE v1.6.11 (62) using the ModelFinder option with 1,000 bootstrap replicates. For each ST, isolates from clinical cases were supplemented with sequences from an Australian cohort of S. aureus bacteremia and publicly available sequences (Table S1).

### Episode-specific variant calling and phylogeny.

To accurately define variants within closely related isolates (i.e., clinical case 1 and 2 *in vitro-*selected mutants), we filtered the output of Snippy as described in reference 19. Briefly, variants were retained only if the read coverage of the index isolate at the same position was ≥10 and the fraction of index reads identical to the reference was >0.5. The curated list of variants was used to generate a phylogenetic tree for the clinical cases using iqtree.

### Episode-specific pangenome.

We used Roary v3.8.2 (63) to generate ortholog clustering from assemblies of closely related isolates (annotated with Prokka v1.12 beta [64]). To exclude genes inaccurately attributed to the accessory genome, we mapped reads to a multi-fasta file of the accessory genes using BWA-MEM v0.7.17-r1188 (65) and scanned the alignment with BEDTools v2.26.0 (66). Only genes for which at least one isolate had a coverage breadth of 0 and at least one isolate had a mean coverage depth of 10 were classified as true accessories.

### Structural variants.

Large deletions were detected by scanning the read alignment using BEDTools. We looked for regions of at least 500 bp with a coverage breadth of 0 in at least one but not all isolates within the group of closely related isolates. To identify smaller insertions and deletions, we analyzed split reads as described in reference 19. The read alignment was scanned for regions with at least 10 split reads in one but not all isolates within the group of related isolates. The genome regions with the primary alignment and the supplementary alignments were annotated and confirmed by manual inspection of the alignment. Structural variants were confirmed by performing a BLAST search of the *de novo* assembly graph of the isolates.

### Genomic and phenotypic analyses of 490 *mec*-negative S. aureus isolates.

We selected 490 *mec*-negative isolates from a combined collection of S. aureus bacteremia isolates (4, 32), supplemented with high-oxacillin-MIC isolates (MIC of ≥1 mg/liter) from a prospective genomic study of multidrug-resistant microorganisms (33). Whole-genome sequencing, quality control, resistome, and multilocus sequence type analysis were performed as described above. Reads were mapped to reference genome BPH2819 (ST5 S. aureus) using Snippy v4.4.3 (https://github.com/tseemann/snippy). A maximum likelihood phylogenetic tree was generated from the core genome alignment of the 490 isolates using iqtree v1.6.11 (67). Oxacillin MICs were assessed using Etest (bioMérieux). The ancestral state of a high oxacillin MIC (MIC of ≥1 mg/liter) as a discrete trait was estimated using the maximum likelihood method implemented in the R package ape v5.4.1 (68).

### GWAS of oxacillin MIC.

A GWAS approach was applied to establish the main core genome determinants of the oxacillin MIC in the S. aureus bacteremia isolates. We first generated a genotype matrix of core genome mutations, excluding synonymous SNPs. Mutations were defined as “core genome” if the position had a coverage of at least 10 reads in all isolates. We then used homoplasyFinder v0.0.0.9000 (69) to determine the consistency index at each locus and kept mutations that had an index of ≤0.5 (indicating at least two independent acquisitions across the phylogeny). We then ran GWAS using the oxacillin MIC binary trait, whereby isolates were categorized as resistant if their oxacillin MIC was ≥1 mg/liter. A Fisher’s exact test was applied to test the association without correcting for the population structure. To correct for the population structure, we used the factored spectrally transformed linear mixed models (FaST-LMM) implemented in pyseer v1.3.6 (70), which computes a kinship matrix based on the core genome SNPs as a random effect. The Bonferroni method was used to correct *P* values for multiple testing.

### Predictive model of high oxacillin MIC.

A machine learning approach based on random forests (71) was applied to establish the ability of the 95 mutations to predict a high oxacillin MIC (MIC of ≥1 mg/liter) in 490 isolates. Using the caret v6.0.86 and ranger v0.12.1 packages in R (72, 73), the model was trained by performing a stratified K-fold cross-validation on the training set (60% of the data sampled by stratifying according to high oxacillin MIC), and the performance of the final model was assessed on the testing set. Model tuning was performed across three sampling strategies (unbalanced, oversampling of resistant strains, and undersampling of sensitive strains), using both Kappa and the area under the receiver operating characteristics (ROC) curve as metrics and using mean decrease impurity (Gini index) and the corrected impurity importance (74) as the variable importance measure. The final model was selected based on the area under the curve. The R code used for splitting the data set, training the model, and assessing performance is provided in Text S1 in the supplemental material.

10.1128/mBio.02882-20.1 TEXT S1 R markdown file with the code used to train and test the random forest models of high oxacillin MIC. Download Text S1, PDF file, 0.2 MB. Copyright © 2020 Giulieri et al. 2020 Giulieri et al. This content is distributed under the terms of the Creative Commons Attribution 4.0 International license.

### Reconstruction of clinical mutations by allelic exchange.

Selected mutations found in clinical isolates and a β-lactamase knockout mutant were constructed using the shuttle plasmid pIMAY-Z as described previously (75). To construct *rpoB* and *pbp3* mutations on the AUS0325 background, alleles carrying the *rpoB*-477V I527V and *pbp3*-V613A mutations were amplified from clinical strain AUS0328 by PCR using primers with tails added. (The primers used in this study are listed in Table S4 in the supplemental material.) Similarly, the *gdpP* wild-type allele was amplified from strain 21162, and the *gdpP* gene carrying the Q572 frameshift was amplified from strain 21163. The construct with partial deletion of *blaZ* was generated by separate PCR amplification of a sequence including the first 213 bp of *blaZ* and a sequence including the last 194 bp, followed by overlap extension PCR. PCR amplicons were then inserted into pIMAY-Z and transformed into E. coli IM08B (AUS0325 background, ST88) and IM30B (21162 background, ST34) to overcome the S. aureus restriction barrier (75). The prepared plasmid was transformed into electrocompetent AUS0325 or 21162/21163, depending on the experiment. Selection of transformants and integration and loss of the plasmid were achieved using the approach described in reference 75, with the exception of the *rpoB* mutant, where a slightly modified procedure was applied, as described in reference 24. All constructed mutants were sequenced and screened for additional mutations using Snippy.

10.1128/mBio.02882-20.10 TABLE S4 Primers used in this study. Download Table S4, XLSX file, 0.01 MB. Copyright © 2020 Giulieri et al. 2020 Giulieri et al. This content is distributed under the terms of the Creative Commons Attribution 4.0 International license.

### Ethics approval and consent to publication.

No consent for publication was sought for the two clinical cases of secondary increase of oxacillin MIC under treatment, as no identifiable details are provided within the manuscript or in the figures.

### Data availability.

The raw reads of the isolates are available in the European Nucleotide Archive under Bioproject accession no. PRJEB40246 (clinical cases, *in vitro*-adapted isolates, and mutants generated by allelic exchange) and PRJEB27932 (GWAS analysis).
