# Supplementary material for: Comprehensive Genomic Investigation of Adaptive Mutations Driving the Low-Level Oxacillin Resistance Phenotype in Staphylococcus aureus
Source: mBio. 2020 Dec 8;11(6):e02882-20. doi: 10.1128/mBio.02882-20 (PMC7733948; doi:10.1128/mBio.02882-20)
Supplement: FIG S1 [file mBio.02882-20-sf001.pdf]

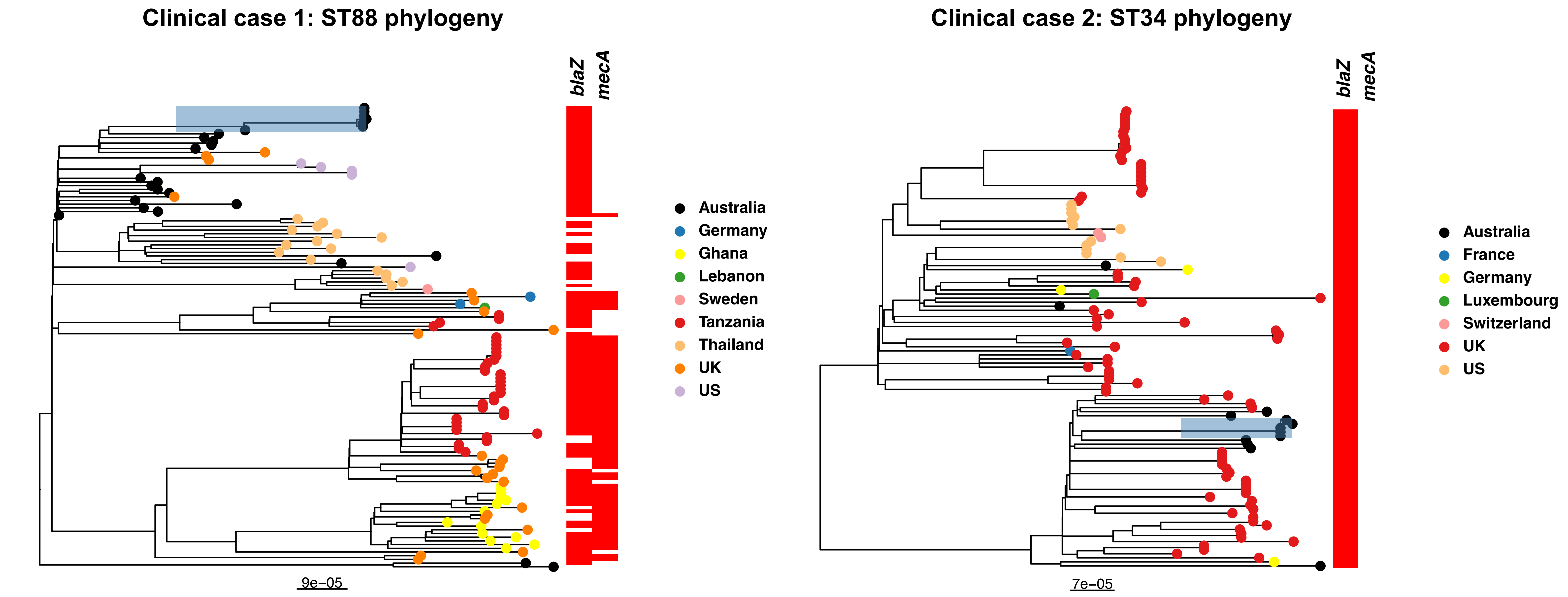

Clinical case 1: ST88 phylogeny
blaZ
mecA
Australia
Germany
Ghana
Lebanon
Sweden
Tanzania
Thailand
UK
US
9e−05
Clinical case 2: ST34 phylogeny
blaZ
mecA
Australia
France
Germany
Luxembourg
Switzerland
UK
US
7e−05
