# Supplementary figures and images for: Comprehensive Genomic Investigation of Adaptive Mutations Driving the Low-Level Oxacillin Resistance Phenotype in Staphylococcus aureus
Source: mBio. 2020 Dec 8;11(6):e02882-20. doi: 10.1128/mBio.02882-20 (PMC7733948; doi:10.1128/mBio.02882-20)

**AUS0325**

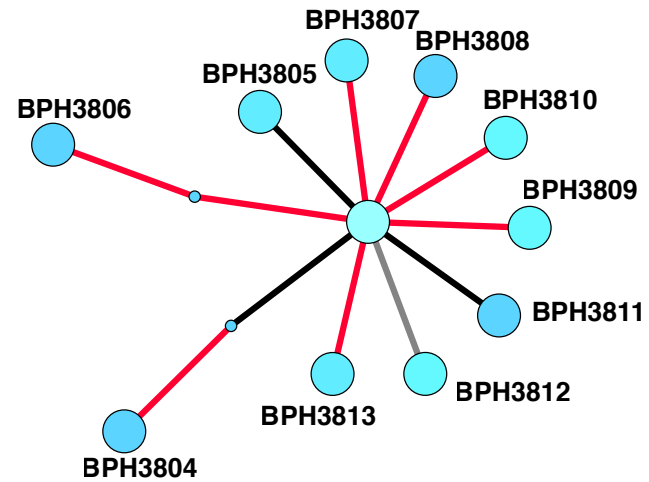

**AUS0325 $\Delta$ blaZ**

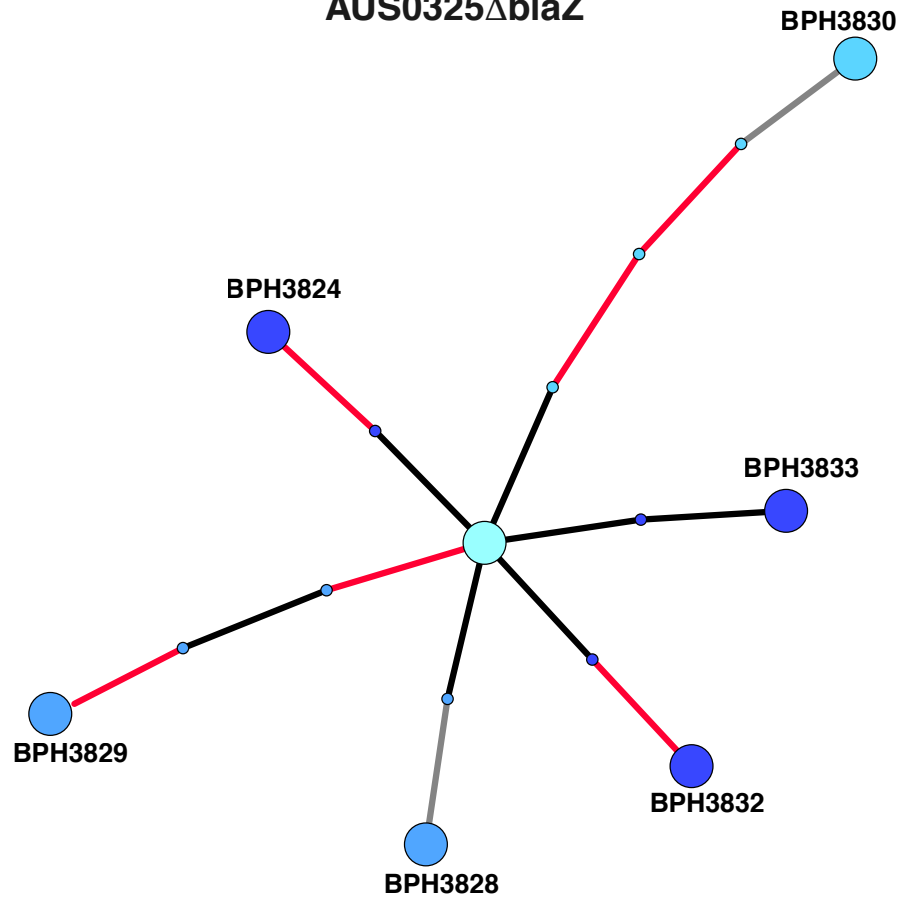

**21162**

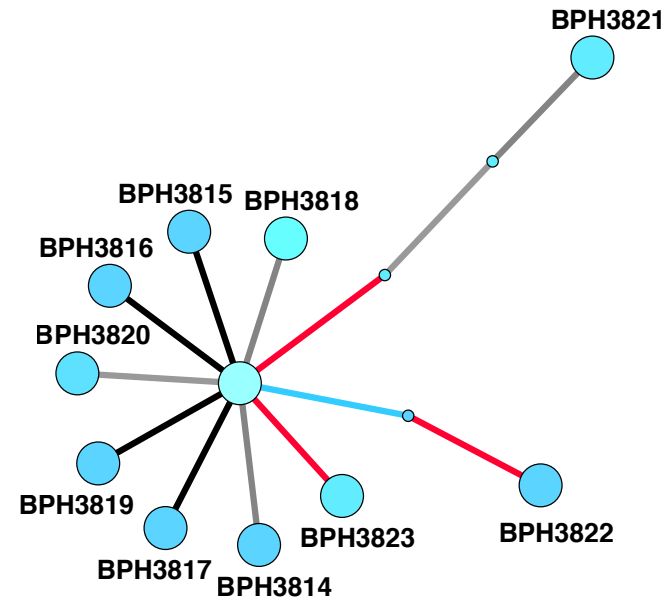

Supplement: FIG S2 [file mBio.02882-20-sf002.pdf]

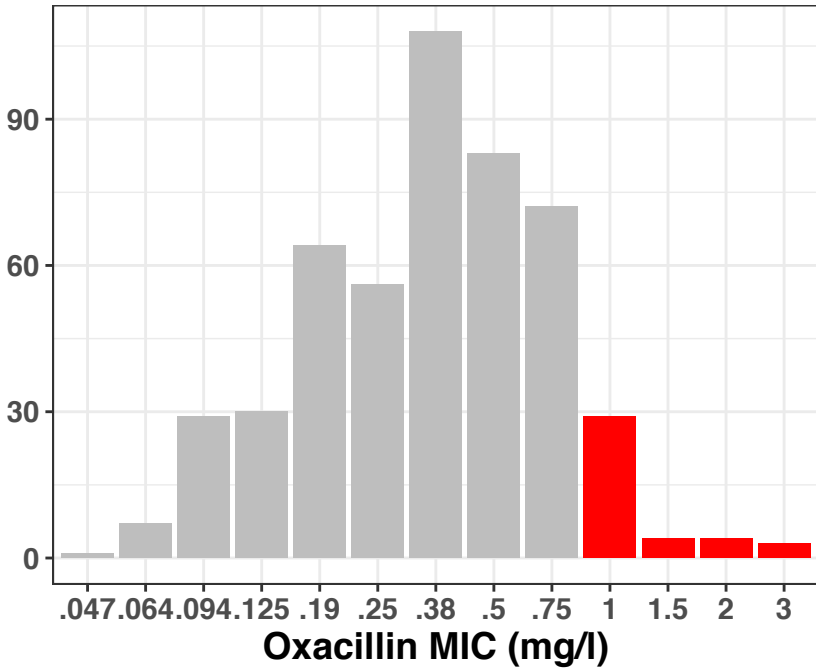

Supplement: FIG S3 [file mBio.02882-20-sf003.pdf]

Performance of the model with the highest AUC when trained on the training dataset

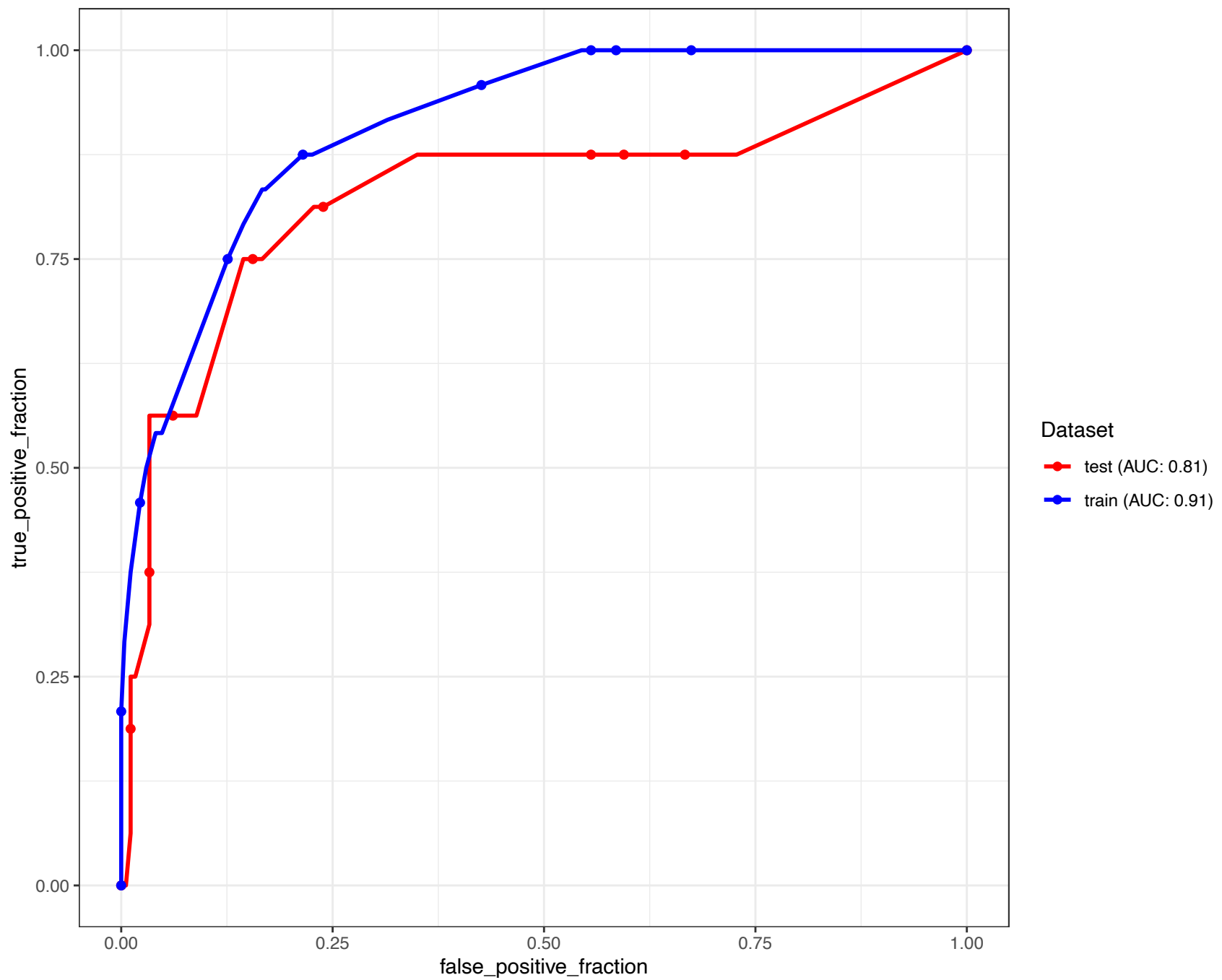

Supplement: FIG S4 [file mBio.02882-20-sf004.pdf]
