# Supplementary material for: Comprehensive Genomic Investigation of Adaptive Mutations Driving the Low-Level Oxacillin Resistance Phenotype in Staphylococcus aureus
Source: mBio. 2020 Dec 8;11(6):e02882-20. doi: 10.1128/mBio.02882-20 (PMC7733948; doi:10.1128/mBio.02882-20)
Supplement: FIG S5 [file mBio.02882-20-sf005.pdf]

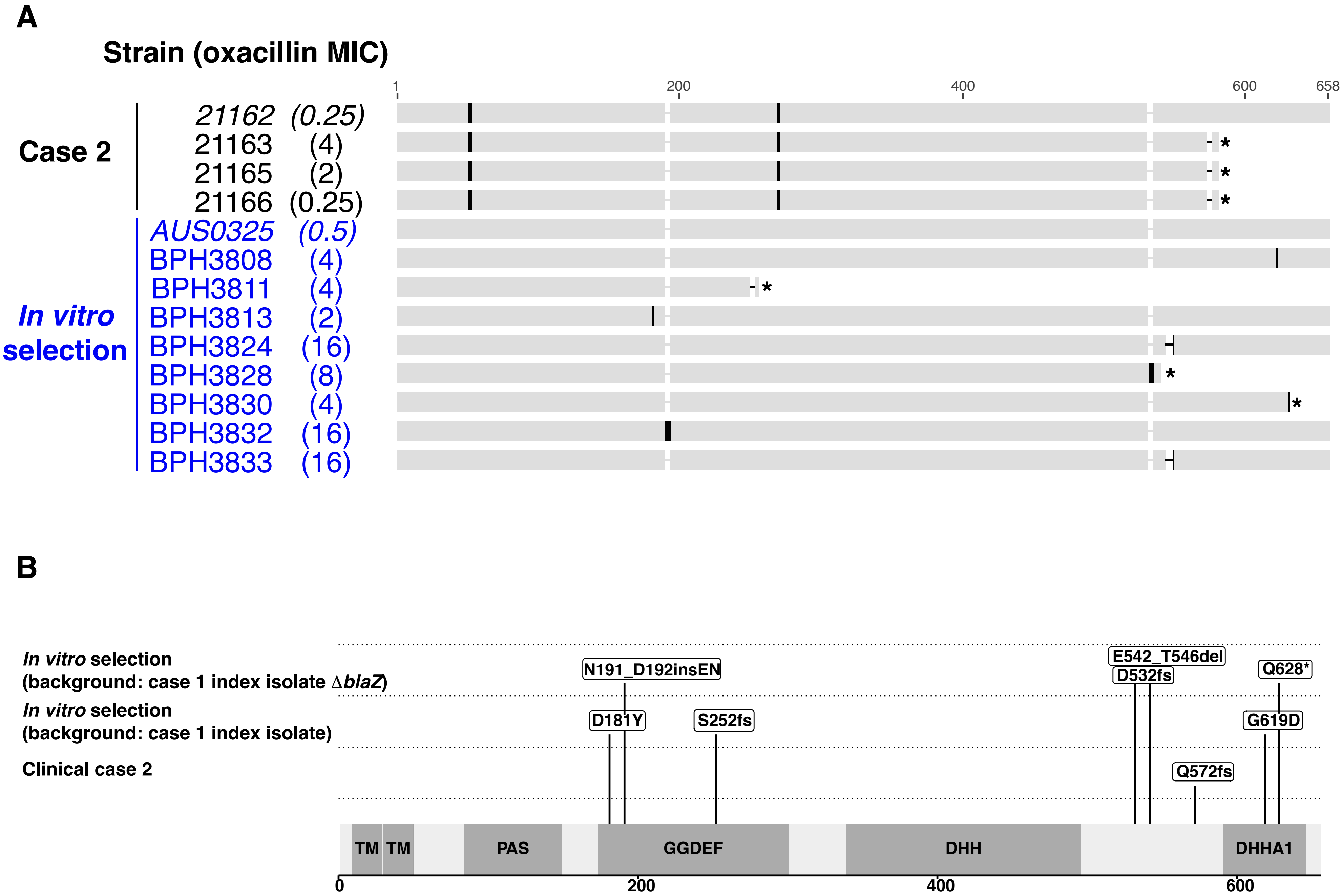
A
Strain (oxacillin MIC)
1
200
400
600
658
Case 2
21162 (0.25)
21163 (4)
21165 (2)
21166 (0.25)
In vitro selection
AUS0325 (0.5)
BPH3808 (4)
BPH3811 (4)
BPH3813 (2)
BPH3824 (16)
BPH3828 (8)
BPH3830 (4)
BPH3832 (16)
BPH3833 (16)
B
In vitro selection (background: case 1 index isolate ΔblaZ)
In vitro selection (background: case 1 index isolate)
Clinical case 2
N191_D192insEN
E542_T546del
D532fs
Q628*
D181Y
S252fs
G619D
Q572fs
TM
TM
PAS
GGDEF
DHH
DHHA1
0
200
400
600
