# Supplementary material for: Comprehensive Genomic Investigation of Adaptive Mutations Driving the Low-Level Oxacillin Resistance Phenotype in Staphylococcus aureus
Source: mBio. 2020 Dec 8;11(6):e02882-20. doi: 10.1128/mBio.02882-20 (PMC7733948; doi:10.1128/mBio.02882-20)
Supplement: TEXT S1 [file mBio.02882-20-s0001.pdf]

Machine learning with stratified cross-validation: oxacillin as binary variable

Stefano Giulieri

05/10/2020

```r
library(tidyverse)
library(magrittr)
library(ranger)
library(caret)
library(tictoc)
library(yardstick)
library(pROC)
```

Import raw data

Mutations in long format

```r
df_simple <- read_tsv("raw_data/mutations.filtered.long.tab")
glimpse(df_simple)
```

```
## Rows: 2,585,240
## Columns: 3
## $ ISOLATE         <chr> "BPH2702", "BPH2702", "BPH2702", "BPH2702", "BPH270...
## $ mutation_id     <chr> "MUT-00004", "MUT-00008", "MUT-00010", "MUT-00011",...
## $ mutation_status <dbl> 0, 1, 0, 0, 0, 0, 0, 0, 0, 0, 0, 0, 0, 0, 0, 0, 0, ...
```

```r
n_distinct(df_simple$ISOLATE)
```

```
## [1] 490
```

```r
n_distinct(df_simple$mutation_id)
```

```
## [1] 5276
```

Phenotype

```r
df_pheno <- read_csv("raw_data/oxacillin_MIC_joined.csv")
glimpse(df_pheno)
```

```
## Rows: 490
## Columns: 3
## $ sample_id  <chr> "BPH2702", "BPH2703", "BPH2706", "BPH2707", "BPH2708", "...
## $ oxamic     <dbl> 0.500, 0.250, 0.125, 0.250, 0.380, 0.190, 0.380, 0.047, ...
## $ oxa_status <chr> "Susceptible", "Susceptible", "Susceptible", "Susceptibl...
```

Annotated GWAS output

```r
df_binary_mixed <- read_csv("raw_data/pyseer_binary_mixed_model.csv")
glimpse(df_binary_mixed)
```

```
## Rows: 5,638
## Columns: 21
## $ variant                      <chr> "MUT-00004", "MUT-00008", "MUT-00010", "...
## $ af                           <dbl> 0.00408, 0.42700, 0.00408, 0.00408, 0.00...
## $ p_value_mixed                <dbl> 0.7720, 0.2880, 0.7720, 0.7720, 0.6880, ...
## $ or_mixed                     <dbl> 0.9433669, 0.9594453, 0.9433669, 0.94336...
## $ bonf_signif_mixed            <lgl> FALSE, FALSE, FALSE, FALSE, FALSE, FALSE...
## $ CHROM                        <chr> "NZ_LR130511", "NZ_LR130511", "NZ_LR1305...
## $ POS                          <dbl> 908, 1408, 1760, 1868, 2003, 3726, 4054,...
## $ REF                          <chr> "C", "A", "C", "G", "G", "C", "C", "G", ...
## $ ALT                          <chr> "T", "G", "T", "A", "A", "A", "T", "A", ...
## $ EFFECT                       <chr> "missense_variant c.908C>T p.Pro303Leu",...
## $ LOCUS_TAG                    <chr> "EW030_RS00005", NA, "EW030_RS00010", "E...
## $ GENE                         <chr> "dnaA", NA, "dnaN", "dnaN", "dnaN", "rec...
## $ `pan gene symbol`            <chr> "dnaA", NA, "dnaN", "dnaN", "dnaN", "rec...
## $ PRODUCT                      <chr> "chromosomal replication initiator prote...
## $ ISOLATE_with_mutation        <chr> "BPH2756##BPH3404", "BPH2702##BPH2703##B...
## $ TYPE                         <chr> "snp", "snp", "snp", "snp", "snp", "snp"...
## $ n_with                       <dbl> 2, 209, 2, 2, 2, 22, 2, 47, 50, 18, 18, ...
## $ ConsistencyIndex             <dbl> 0.5000000, 0.1428571, 0.5000000, 0.50000...
## $ Counts                       <chr> "488:02:00", "281:209", "488:02:00", "48...
## $ MinimumNumberChangesOnTree <dbl> 2, 7, 2, 2, 2, NA, 2, 3, 2, 3, 3, 2, 3, ...
## $ ST_with                      <chr> "567|2605", "15|8|97|NA|109|20|1|88|12|1...
```

```r
# create mutations labels
df_binary_mixed <- df_binary_mixed %>%
   mutate(mutation_label = case_when(`pan gene symbol` != "-" ~ `pan gene symbol`,
                                     !is.na(LOCUS_TAG) ~ LOCUS_TAG,
                                     TRUE ~ "intergenic")) %>%
  mutate(mutation_label = str_c(TYPE, POS, mutation_label, sep = "_")) %>%
  mutate(direction = if_else(or_mixed > 1, "+", "-")) %>%
  relocate(mutation_label, direction, .after = variant)

df_binary_mixed %>%
  select(variant, mutation_label, direction)
```

```
## # A tibble: 5,638 x 3
##    variant   mutation_label      direction
##    <chr>     <chr>               <chr>
##  1 MUT-00004 snp_908_dnaA        -
##  2 MUT-00008 snp_1408_intergenic -
##  3 MUT-00010 snp_1760_dnaN       -
##  4 MUT-00011 snp_1868_dnaN       -
##  5 MUT-00012 snp_2003_dnaN       -
##  6 MUT-00017 snp_3726_recF       +
##  7 MUT-00019 snp_4054_recF       -
##  8 MUT-00021 snp_4110_recF       -
##  9 MUT-00024 snp_4329_recF       +
## 10 MUT-00026 snp_4957_gyrB       -
## # ... with 5,628 more rows
```

Genotype matrix: only mutations above the Bonferroni-corrected significance threshold

```
signif_mutations <- df_binary_mixed %>%
  filter(bonf_signif_mixed) %>%
  .$variant

df_geno <- df_simple %>%
  filter(mutation_id %in% signif_mutations) %>%
  pivot_wider(names_from = mutation_id, values_from = mutation_status)

snps <- df_geno %>%
  column_to_rownames("ISOLATE") %>%
  as.matrix()
str(snps)
```

```
##  num [1:490, 1:95] 1 1 1 1 1 1 1 1 1 1 ...
##  - attr(*, "dimnames")=List of 2
##   ..$ : chr [1:490] "BPH2702" "BPH2703" "BPH2706" "BPH2707" ...
##   ..$ : chr [1:95] "MUT-00231" "MUT-00232" "MUT-00315" "MUT-00381" ...
```

Phenotype vector

Oxacillin as binary variable

```
phen <- as.factor(df_pheno$oxa_status)
names(phen) <- df_pheno$sample_id
str(phen)
```

```
##  Factor w/ 2 levels "Resistant","Susceptible": 2 2 2 2 2 2 2 2 2 2 ...
##  - attr(*, "names")= chr [1:490] "BPH2702" "BPH2703" "BPH2706" "BPH2707" ...
```

```
table(phen)
```

```
## phen
##   Resistant Susceptible
##          40         450
```

Partition the data

```
set.seed(1234)
trainIndex <- createDataPartition(phen, p = .6,
                                  list = FALSE,
                                  times = 1)

# train dataset
x_train <- snps[trainIndex,]
str(x_train)
```

```
##  num [1:294, 1:95] 1 1 1 1 1 1 1 1 1 0 ...
##  - attr(*, "dimnames")=List of 2
##   ..$ : chr [1:294] "BPH2703" "BPH2707" "BPH2711" "BPH2717" ...
##   ..$ : chr [1:95] "MUT-00231" "MUT-00232" "MUT-00315" "MUT-00381" ...
```

```
y_train <- phen[trainIndex]
str(y_train)
```

```
##  Factor w/ 2 levels "Resistant","Susceptible": 2 2 2 2 2 2 2 2 2 2 ...
##  - attr(*, "names")= chr [1:294] "BPH2703" "BPH2707" "BPH2711" "BPH2717" ...
```

```
table(y_train)
```

```
## y_train
##   Resistant Susceptible
##          24         270
```

```
# test dataset
x_test <- snps[-trainIndex,]
str(x_test)
```

```
##  num [1:196, 1:95] 1 1 1 1 1 1 1 0 1 1 ...
##  - attr(*, "dimnames")=List of 2
##   ..$ : chr [1:196] "BPH2702" "BPH2706" "BPH2708" "BPH2712" ...
##   ..$ : chr [1:95] "MUT-00231" "MUT-00232" "MUT-00315" "MUT-00381" ...
```

```
y_test <- phen[-trainIndex]
str(y_test)
```

```
##  Factor w/ 2 levels "Resistant","Susceptible": 2 2 2 2 2 2 2 2 2 2 ...
##  - attr(*, "names")= chr [1:196] "BPH2702" "BPH2706" "BPH2708" "BPH2712" ...
```

```
table(y_test)
```

```
## y_test
##   Resistant Susceptible
##          16         180
```

Train the model - using 3 different sampling strategies

While caret searches RF hyperparameters for us, we need to create a mechanism to loop through different sampling strategies (unbalanced: no correction for the inbalance between cases and controls; up: increase sampling of cases; down" decreased sampling of controls), the metric used (here: ROC or kappa, since the latter is better if there is an inbalance between cases and controls). Finally we compare variable importance measured with the Gini index ("impurity") with a modified version of the Gini index that was proposed here (https://academic.oup.com/bioinformatics/article/34/21/3711/4994791) and that should correct the bias associated with the Gini index in genetic studies and is not as computionally intensive as permutation.

```
# function to train the model using k=10 cross-validation, stratified by the binary outcome (high oxaci

train_model <- function(sampling, # sampling mode
                        metric = "ROC",
                        importance = "impurity"){ # impurity (Gini index) is default metric. Alternativ

  message(glue::glue("Fitting ranger model using sampling strategy: {sampling}, metric: {metric}, impor

  # fix sampling argument when sampling is "unbalanced"
 if (sampling == "unbalanced") {
   my_sampling <- NULL
 } else {
   my_sampling <- sampling
 }

  # generate dataset indexes for stratified cross-validation
  index <- createFolds(y_train, k = 10, list = T, returnTrain = T)

  # prepare cross-validation
  if (metric != "ROC"){
    train_control <- trainControl(method = "cv",
                      number = 10,
                      index = index,
                      classProbs = T,
                      verboseIter = F,
                      sampling = my_sampling)
  } else {
     train_control <- trainControl(method = "cv",
                      number = 10,
                      index = index,
                      summaryFunction = twoClassSummary,
                      classProbs = TRUE,
                      verboseIter = F,
                      sampling = my_sampling)
  }
```

```r
# train the model
set.seed(1234)
tic("Model fitting started:")
m_train <- train(x = x_train,
                 y = y_train,
                  method = "ranger",
                  metric = metric,
                 importance = importance,
                  trControl = train_control,
                 tuneLength = 5)
toc()

# construct dataframe of feature importance
importance <- m_train$finalModel$variable.importance
variant <- names(m_train$finalModel$variable.importance)

df_features <- tibble(variant, importance)

out <- list(m_train, df_features)
names <- str_c(sampling_mode, metric, importance, sep = "_")

return(out)

}

sampling_mode <- c("unbalanced", "up", "down")
metric <- c("ROC", "Kappa")
importance <- c("impurity", "impurity_corrected")
args <- expand_grid(sampling_mode, metric, importance) %>%
  unite(args) %>%
  .$args

models <- lapply(args, function(x) {
  sampling <- str_split_fixed(x, "_", 3)[1]
  metric <- str_split_fixed(x, "_", 3)[2]
  importance <- str_split_fixed(x, "_", 3)[3]
  train_model(sampling = sampling, metric = metric, importance = importance)
})
```

```
## Fitting ranger model using sampling strategy: unbalanced, metric: ROC, importance variable: impurity

## Model fitting started:: 11.899 sec elapsed

## Fitting ranger model using sampling strategy: unbalanced, metric: ROC, importance variable: impurity_

## Model fitting started:: 13.172 sec elapsed

## Fitting ranger model using sampling strategy: unbalanced, metric: Kappa, importance variable: impuri

## Model fitting started:: 11.789 sec elapsed
```

```
## Fitting ranger model using sampling strategy: unbalanced, metric: Kappa, importance variable: impuri

## Model fitting started:: 13.091 sec elapsed

## Fitting ranger model using sampling strategy: up, metric: ROC, importance variable: impurity

## Model fitting started:: 18.375 sec elapsed

## Fitting ranger model using sampling strategy: up, metric: ROC, importance variable: impurity_correct

## Model fitting started:: 30.681 sec elapsed

## Fitting ranger model using sampling strategy: up, metric: Kappa, importance variable: impurity

## Model fitting started:: 17.764 sec elapsed

## Fitting ranger model using sampling strategy: up, metric: Kappa, importance variable: impurity_corre

## Model fitting started:: 28.778 sec elapsed

## Fitting ranger model using sampling strategy: down, metric: ROC, importance variable: impurity

## Model fitting started:: 5.063 sec elapsed

## Fitting ranger model using sampling strategy: down, metric: ROC, importance variable: impurity_corre

## Model fitting started:: 5.307 sec elapsed

## Fitting ranger model using sampling strategy: down, metric: Kappa, importance variable: impurity

## Model fitting started:: 5.378 sec elapsed

## Fitting ranger model using sampling strategy: down, metric: Kappa, importance variable: impurity_cor

## Model fitting started:: 5.295 sec elapsed
```

```r
# assign names and fix long names
args_short <- str_replace(args, "unbalanced", "unbal") %>% str_replace("Kappa", "k") %>% str_replace("i
df_args <- tibble(args, args_short)
names(models) <- args_short

models_simplified <- lapply(models, function(x) x[[1]])
print(models_simplified)
```

```
## $unbal_ROC_impurity
## Random Forest
##
## 294 samples
##  95 predictor
##   2 classes: 'Resistant', 'Susceptible'
##
## No pre-processing
## Resampling: Cross-Validated (10 fold)
## Summary of sample sizes: 265, 265, 265, 264, 264, 264, ...
## Resampling results across tuning parameters:
##
##   mtry  splitrule   ROC        Sens        Spec
##    2    gini        0.7608025  0.00000000  0.9888889
##    2    extratrees  0.7675926  0.00000000  0.9814815
##   25    gini        0.7577160  0.03333333  0.9777778
##   25    extratrees  0.7521605  0.11666667  0.9777778
##   48    gini        0.7496914  0.11666667  0.9777778
##   48    extratrees  0.7462963  0.11666667  0.9777778
##   71    gini        0.7370370  0.08333333  0.9777778
##   71    extratrees  0.7425926  0.13333333  0.9814815
##   95    gini        0.7280864  0.11666667  0.9851852
##   95    extratrees  0.7336420  0.16666667  0.9814815
##
## Tuning parameter 'min.node.size' was held constant at a value of 1
## ROC was used to select the optimal model using the largest value.
## The final values used for the model were mtry = 2, splitrule = extratrees
##  and min.node.size = 1.
##
## $unbal_ROC_impurity_c
## Random Forest
##
## 294 samples
##  95 predictor
##   2 classes: 'Resistant', 'Susceptible'
##
## No pre-processing
## Resampling: Cross-Validated (10 fold)
## Summary of sample sizes: 265, 264, 265, 265, 264, 265, ...
## Resampling results across tuning parameters:
##
##   mtry  splitrule   ROC        Sens       Spec
##    2    gini        0.7947531  0.0000000  0.9962963
##    2    extratrees  0.7712963  0.0000000  1.0000000
##   25    gini        0.7706790  0.2333333  0.9296296
##   25    extratrees  0.8021605  0.3166667  0.9592593
##   48    gini        0.7527778  0.2833333  0.9703704
##   48    extratrees  0.7620370  0.2000000  0.9814815
##   71    gini        0.7283951  0.1666667  0.9444444
##   71    extratrees  0.7027778  0.2166667  0.9629630
##   95    gini        0.7089506  0.1666667  0.9851852
##   95    extratrees  0.7274691  0.2000000  0.9666667
##
## Tuning parameter 'min.node.size' was held constant at a value of 1
```

```
## ROC was used to select the optimal model using the largest value.
## The final values used for the model were mtry = 25, splitrule = extratrees
##  and min.node.size = 1.
##
## $unbal_k_impurity
## Random Forest
##
## 294 samples
##  95 predictor
##   2 classes: 'Resistant', 'Susceptible'
##
## No pre-processing
## Resampling: Cross-Validated (10 fold)
## Summary of sample sizes: 265, 264, 265, 265, 264, 265, ...
## Resampling results across tuning parameters:
##
##   mtry  splitrule   Accuracy   Kappa
##    2    gini        0.9082759  0.03941149
##    2    extratrees  0.9082759  0.03941149
##   25    gini        0.9013793  0.07410935
##   25    extratrees  0.9013793  0.07410935
##   48    gini        0.9012644  0.06090179
##   48    extratrees  0.9012644  0.06090179
##   71    gini        0.9081609  0.16527069
##   71    extratrees  0.9048276  0.07892862
##   95    gini        0.9013793  0.07410935
##   95    extratrees  0.9081609  0.16527069
##
## Tuning parameter 'min.node.size' was held constant at a value of 1
## Kappa was used to select the optimal model using the largest value.
## The final values used for the model were mtry = 71, splitrule = gini
##  and min.node.size = 1.
##
## $unbal_k_impurity_c
## Random Forest
##
## 294 samples
##  95 predictor
##   2 classes: 'Resistant', 'Susceptible'
##
## No pre-processing
## Resampling: Cross-Validated (10 fold)
## Summary of sample sizes: 265, 264, 265, 265, 264, 265, ...
## Resampling results across tuning parameters:
##
##   mtry  splitrule   Accuracy   Kappa
##    2    gini        0.9151724  -0.004819277
##    2    extratrees  0.9186207   0.000000000
##   25    gini        0.8709195   0.134071965
##   25    extratrees  0.9047126   0.223742550
##   48    gini        0.9148276   0.301602029
##   48    extratrees  0.9183908   0.219527604
##   71    gini        0.8810345   0.145216223
##   71    extratrees  0.9011494   0.163986564
```

```
##   95    gini        0.9214943   0.205551318
##   95    extratrees  0.9050575   0.191816495
##
## Tuning parameter 'min.node.size' was held constant at a value of 1
## Kappa was used to select the optimal model using the largest value.
## The final values used for the model were mtry = 48, splitrule = gini
##  and min.node.size = 1.
##
## $up_ROC_impurity
## Random Forest
##
## 294 samples
##  95 predictor
##   2 classes: 'Resistant', 'Susceptible'
##
## No pre-processing
## Resampling: Cross-Validated (10 fold)
## Summary of sample sizes: 265, 264, 265, 265, 264, 265, ...
## Addtional sampling using up-sampling
##
## Resampling results across tuning parameters:
##
##   mtry  splitrule   ROC        Sens       Spec
##    2    gini        0.7274691  0.3666667  0.9148148
##    2    extratrees  0.7151235  0.3666667  0.9148148
##   25    gini        0.6972222  0.6000000  0.8111111
##   25    extratrees  0.7126543  0.6000000  0.8296296
##   48    gini        0.6891975  0.6000000  0.8296296
##   48    extratrees  0.6854938  0.6000000  0.8259259
##   71    gini        0.6666667  0.6000000  0.8111111
##   71    extratrees  0.6799383  0.6000000  0.8074074
##   95    gini        0.6898148  0.6000000  0.8185185
##   95    extratrees  0.6888889  0.6000000  0.8074074
##
## Tuning parameter 'min.node.size' was held constant at a value of 1
## ROC was used to select the optimal model using the largest value.
## The final values used for the model were mtry = 2, splitrule = gini
##  and min.node.size = 1.
##
## $up_ROC_impurity_c
## Random Forest
##
## 294 samples
##  95 predictor
##   2 classes: 'Resistant', 'Susceptible'
##
## No pre-processing
## Resampling: Cross-Validated (10 fold)
## Summary of sample sizes: 265, 264, 265, 264, 264, 265, ...
## Addtional sampling using up-sampling
##
## Resampling results across tuning parameters:
##
##   mtry  splitrule   ROC        Sens       Spec
```

```
##    2    gini        0.7453704  0.4666667  0.9185185
##    2    extratrees  0.7175926  0.4500000  0.9222222
##   25    gini        0.7330247  0.6333333  0.8185185
##   25    extratrees  0.7175926  0.5833333  0.8259259
##   48    gini        0.7379630  0.6000000  0.8000000
##   48    extratrees  0.7311728  0.6333333  0.8074074
##   71    gini        0.7391975  0.6333333  0.7481481
##   71    extratrees  0.6898148  0.6166667  0.8259259
##   95    gini        0.6929012  0.5333333  0.7444444
##   95    extratrees  0.7299383  0.5833333  0.7555556
##
## Tuning parameter 'min.node.size' was held constant at a value of 1
## ROC was used to select the optimal model using the largest value.
## The final values used for the model were mtry = 2, splitrule = gini
##  and min.node.size = 1.
##
## $up_k_impurity
## Random Forest
##
## 294 samples
##  95 predictor
##   2 classes: 'Resistant', 'Susceptible'
##
## No pre-processing
## Resampling: Cross-Validated (10 fold)
## Summary of sample sizes: 265, 264, 265, 264, 264, 265, ...
## Addtional sampling using up-sampling
##
## Resampling results across tuning parameters:
##
##   mtry  splitrule   Accuracy   Kappa
##    2    gini        0.8611494  0.2665998
##    2    extratrees  0.8647126  0.3156848
##   25    gini        0.7963218  0.2115799
##   25    extratrees  0.7864368  0.2096797
##   48    gini        0.7898851  0.2111000
##   48    extratrees  0.7933333  0.2175626
##   71    gini        0.7897701  0.2107105
##   71    extratrees  0.7963218  0.2136235
##   95    gini        0.7796552  0.1965312
##   95    extratrees  0.7895402  0.2037770
##
## Tuning parameter 'min.node.size' was held constant at a value of 1
## Kappa was used to select the optimal model using the largest value.
## The final values used for the model were mtry = 2, splitrule = extratrees
##  and min.node.size = 1.
##
## $up_k_impurity_c
## Random Forest
##
## 294 samples
##  95 predictor
##   2 classes: 'Resistant', 'Susceptible'
##
```

```
## No pre-processing
## Resampling: Cross-Validated (10 fold)
## Summary of sample sizes: 265, 264, 265, 264, 264, 265, ...
## Addtional sampling using up-sampling
##
## Resampling results across tuning parameters:
##
##   mtry  splitrule   Accuracy   Kappa
##    2    gini        0.8780460  0.2949980
##    2    extratrees  0.8817241  0.2881341
##   25    gini        0.7998851  0.2481056
##   25    extratrees  0.8035632  0.2440845
##   48    gini        0.7790805  0.1897446
##   48    extratrees  0.7900000  0.2364195
##   71    gini        0.7354023  0.1768670
##   71    extratrees  0.8064368  0.2454449
##   95    gini        0.7254023  0.1531199
##   95    extratrees  0.7381609  0.1771179
##
## Tuning parameter 'min.node.size' was held constant at a value of 1
## Kappa was used to select the optimal model using the largest value.
## The final values used for the model were mtry = 2, splitrule = gini
##  and min.node.size = 1.
##
## $down_ROC_impurity
## Random Forest
##
## 294 samples
##  95 predictor
##   2 classes: 'Resistant', 'Susceptible'
##
## No pre-processing
## Resampling: Cross-Validated (10 fold)
## Summary of sample sizes: 265, 264, 265, 264, 264, 265, ...
## Addtional sampling using down-sampling
##
## Resampling results across tuning parameters:
##
##   mtry  splitrule   ROC        Sens       Spec
##    2    gini        0.7731481  0.4833333  0.8777778
##    2    extratrees  0.7731481  0.5500000  0.8777778
##   25    gini        0.7737654  0.7000000  0.7777778
##   25    extratrees  0.7391975  0.6166667  0.7629630
##   48    gini        0.7234568  0.7000000  0.7370370
##   48    extratrees  0.7543210  0.6666667  0.7851852
##   71    gini        0.7641975  0.7333333  0.7555556
##   71    extratrees  0.7274691  0.7333333  0.7296296
##   95    gini        0.7092593  0.7333333  0.7111111
##   95    extratrees  0.6725309  0.7000000  0.7518519
##
## Tuning parameter 'min.node.size' was held constant at a value of 1
## ROC was used to select the optimal model using the largest value.
## The final values used for the model were mtry = 25, splitrule = gini
##  and min.node.size = 1.
```

```
##
## $down_ROC_impurity_c
## Random Forest
##
## 294 samples
##  95 predictor
##   2 classes: 'Resistant', 'Susceptible'
##
## No pre-processing
## Resampling: Cross-Validated (10 fold)
## Summary of sample sizes: 265, 265, 264, 265, 264, 264, ...
## Addtional sampling using down-sampling
##
## Resampling results across tuning parameters:
##
##   mtry  splitrule   ROC        Sens       Spec
##    2    gini        0.7336420  0.4500000  0.8888889
##    2    extratrees  0.7706790  0.3666667  0.8592593
##   25    gini        0.6861111  0.6333333  0.7444444
##   25    extratrees  0.7104938  0.6333333  0.6814815
##   48    gini        0.6879630  0.5833333  0.7037037
##   48    extratrees  0.7435185  0.6666667  0.7666667
##   71    gini        0.6891975  0.6833333  0.6259259
##   71    extratrees  0.6756173  0.4833333  0.7481481
##   95    gini        0.7391975  0.6666667  0.7555556
##   95    extratrees  0.6376543  0.4166667  0.7370370
##
## Tuning parameter 'min.node.size' was held constant at a value of 1
## ROC was used to select the optimal model using the largest value.
## The final values used for the model were mtry = 2, splitrule = extratrees
##  and min.node.size = 1.
##
## $down_k_impurity
## Random Forest
##
## 294 samples
##  95 predictor
##   2 classes: 'Resistant', 'Susceptible'
##
## No pre-processing
## Resampling: Cross-Validated (10 fold)
## Summary of sample sizes: 265, 265, 264, 265, 264, 264, ...
## Addtional sampling using down-sampling
##
## Resampling results across tuning parameters:
##
##   mtry  splitrule   Accuracy   Kappa
##    2    gini        0.8439080  0.2077478
##    2    extratrees  0.8640230  0.3106056
##   25    gini        0.7485057  0.1961937
##   25    extratrees  0.7457471  0.2303129
##   48    gini        0.7585057  0.2475734
##   48    extratrees  0.7306897  0.1805819
##   71    gini        0.7306897  0.1885948
```

```
##   71    extratrees  0.7357471  0.1546487
##   95    gini        0.7280460  0.1942325
##   95    extratrees  0.7243678  0.1390263
##
## Tuning parameter 'min.node.size' was held constant at a value of 1
## Kappa was used to select the optimal model using the largest value.
## The final values used for the model were mtry = 2, splitrule = extratrees
##  and min.node.size = 1.
##
## $down_k_impurity_c
## Random Forest
##
## 294 samples
##  95 predictor
##   2 classes: 'Resistant', 'Susceptible'
##
## No pre-processing
## Resampling: Cross-Validated (10 fold)
## Summary of sample sizes: 265, 265, 264, 265, 264, 264, ...
## Addtional sampling using down-sampling
##
## Resampling results across tuning parameters:
##
##   mtry  splitrule   Accuracy   Kappa
##    2    gini        0.8535632  0.2671622
##    2    extratrees  0.8196552  0.1324897
##   25    gini        0.7355172  0.2015882
##   25    extratrees  0.6754023  0.1440706
##   48    gini        0.6940230  0.1774022
##   48    extratrees  0.7581609  0.2409920
##   71    gini        0.6298851  0.1142251
##   71    extratrees  0.7275862  0.1146897
##   95    gini        0.7483908  0.2182373
##   95    extratrees  0.7109195  0.1123600
##
## Tuning parameter 'min.node.size' was held constant at a value of 1
## Kappa was used to select the optimal model using the largest value.
## The final values used for the model were mtry = 2, splitrule = gini
##  and min.node.size = 1.
```

Extract variable importance of the different models

```
list <- list()

for (i in 1:length(models)){
  df <- models[[i]][[2]] %>%
    mutate(model = names(models)[i]) %>%
    separate(model, into = c("sampling", "metric", "importance_type"), sep = "_", remove = F, extra = "

  # confirm method
```

```
  print(models_simplified[[i]]$finalModel$importance.mode)

  list[[i]] <- df

}
```

```
## [1] "impurity"
## [1] "impurity_corrected"
## [1] "impurity"
## [1] "impurity_corrected"
## [1] "impurity"
## [1] "impurity_corrected"
## [1] "impurity"
## [1] "impurity_corrected"
## [1] "impurity"
## [1] "impurity_corrected"
## [1] "impurity"
## [1] "impurity_corrected"
```

```
df_features <- bind_rows(list) %>%
  left_join(df_binary_mixed)
```

```
## Joining, by = "variant"
```

```
rm(list)
```

Assess model perfomance on the train dataset

```
# dataframe based on prediction probabilities (for ROC curves)
roclist <- list()

for (i in 1:length(models_simplified)) {
  m <- models_simplified[[i]]
  s <- names(models_simplified)[i]

  pred1 <- predict(m, newdata = x_train, type = "prob")

  pred2 <- predict(m, newdata = x_train)

  df_roc <- tibble(
    truth = y_train,
    pred_resistant = pred1$Resistant,
    pred_susceptible = pred1$Susceptible,
    sample_id = rownames(x_train),
    model = s,
    predicted = pred2
  ) %>%
    mutate(y_train_binary = if_else(y_train == "Resistant", 1, 0))
```

```r
  roclist[[i]] <- df_roc

}

df_roc_train <- bind_rows(roclist)
rm(roclist)

# dataframe based on binary prediction
metrics_list <- list()

for (i in 1:length(models_simplified)){
  s <- names(models_simplified)[i]
  data <- df_roc_train %>%
    filter(model == s)

   cm <- yardstick::conf_mat(data, truth = truth, predicted)




   auc <- data %>%
     yardstick::roc_auc(truth, pred_resistant)

  metrics <- summary(cm) %>%
    add_row(auc) %>%
    mutate(model = s)



  metrics_list[[i]] <- metrics
}

df_metrics_train <- bind_rows(metrics_list)
rm(metrics_list)

# plot metrics
df_metrics_train %>%
  ggplot(aes(x = .metric, y = .estimate, fill = model)) +
  geom_bar(stat = "identity", position = "dodge") +
  scale_fill_viridis_d() +
  coord_flip() +
  theme_bw()
```

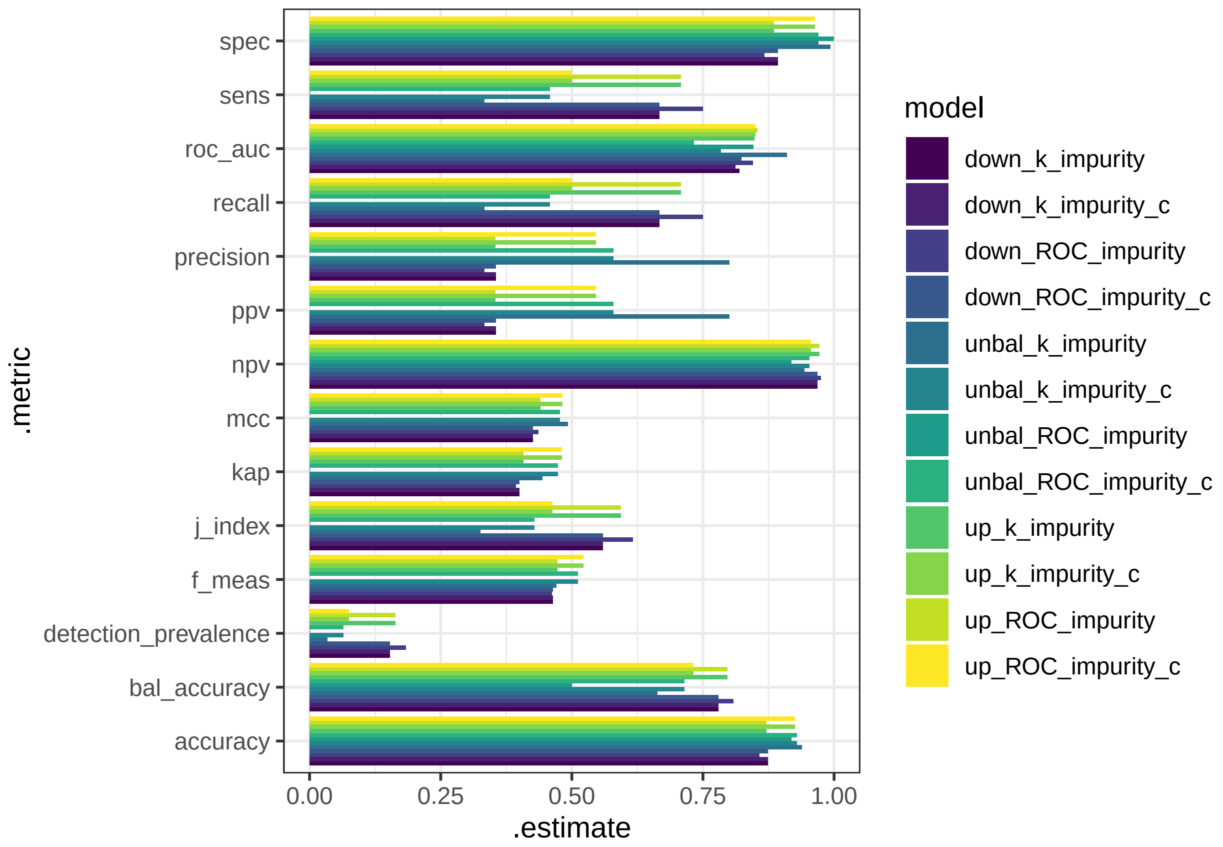


```
# plot roc curves
ggplot(df_roc_train) +
  plotROC::geom_roc(aes(d = y_train_binary, m = pred_resistant, colour = model),labels = F) +
 scale_color_viridis_d(guide = F) +
  geom_text(data = df_metrics_train %>% filter(.metric == "roc_auc"), aes(label = str_c("AUC: ", round(
  facet_wrap(~model) +
   theme_bw()
```

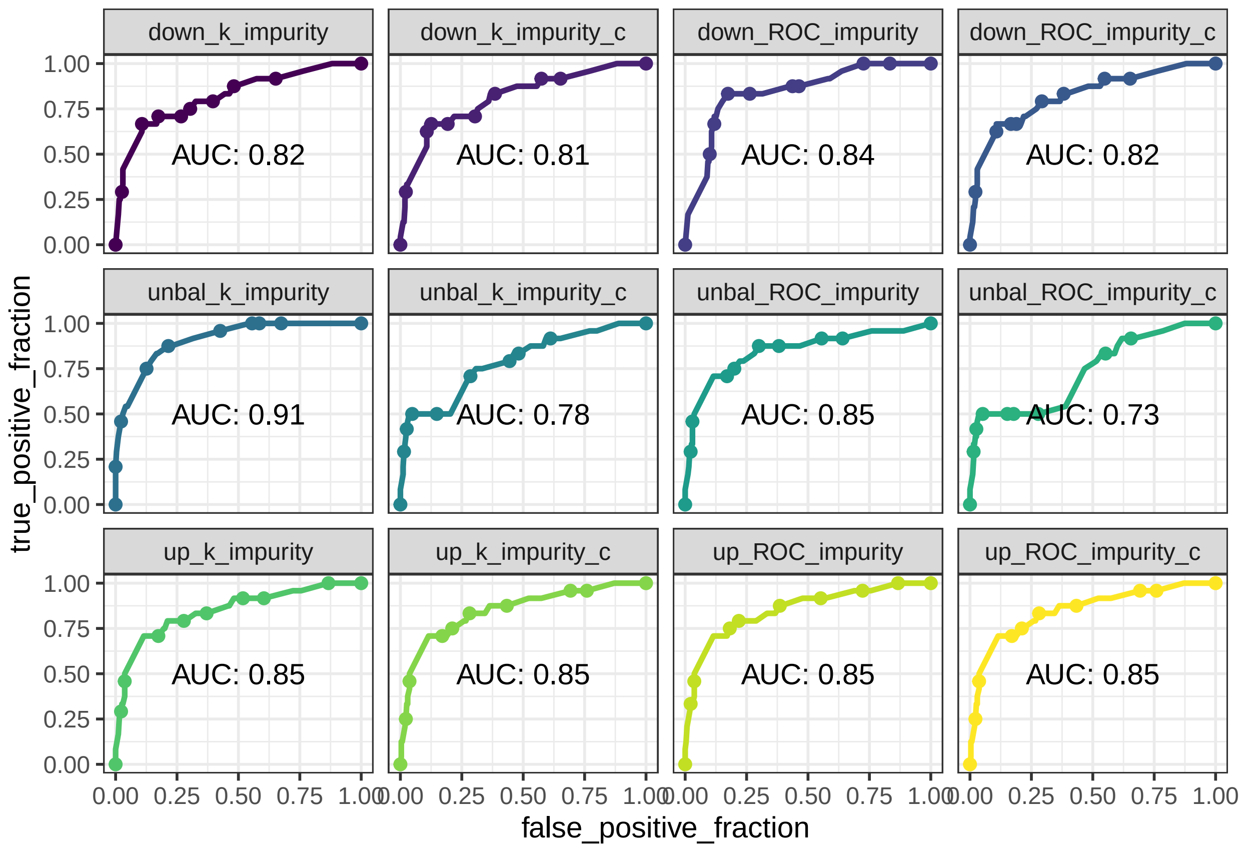

Conclusion

The model with the highest AUC is

```
highest_auc <- df_metrics_train %>% filter(.metric == "roc_auc") %>% slice_max(order_by = .estimate, n
highest_auc
```

```
## [1] "unbal_k_impurity"
```

Model performance in the test dataset

```
s <- highest_auc
m <- models[[which(names(models) == s)]]
m <- m[[1]]
m
```

```
## Random Forest
##
## 294 samples
##  95 predictor
##   2 classes: 'Resistant', 'Susceptible'
```

```
##
## No pre-processing
## Resampling: Cross-Validated (10 fold)
## Summary of sample sizes: 265, 264, 265, 265, 264, 265, ...
## Resampling results across tuning parameters:
##
##   mtry  splitrule   Accuracy   Kappa
##    2    gini        0.9082759  0.03941149
##    2    extratrees  0.9082759  0.03941149
##   25    gini        0.9013793  0.07410935
##   25    extratrees  0.9013793  0.07410935
##   48    gini        0.9012644  0.06090179
##   48    extratrees  0.9012644  0.06090179
##   71    gini        0.9081609  0.16527069
##   71    extratrees  0.9048276  0.07892862
##   95    gini        0.9013793  0.07410935
##   95    extratrees  0.9081609  0.16527069
##
## Tuning parameter 'min.node.size' was held constant at a value of 1
## Kappa was used to select the optimal model using the largest value.
## The final values used for the model were mtry = 71, splitrule = gini
##  and min.node.size = 1.
```

```
pred1 <- predict(m, newdata = x_test, type = "prob")

pred2 <- predict(m, newdata = x_test)

df_roc_test <- tibble(
    truth = y_test,
    pred_resistant = pred1$Resistant,
    pred_susceptible = pred1$Susceptible,
    sample_id = rownames(x_test),
    model = s,
    predicted = pred2
  ) %>%
    mutate(y_test_binary = if_else(y_test == "Resistant", 1, 0))

cm_test <- yardstick::conf_mat(df_roc, truth = truth, predicted)

p <- autoplot(cm, type = "heatmap") +
    ggtitle(s)

p
```

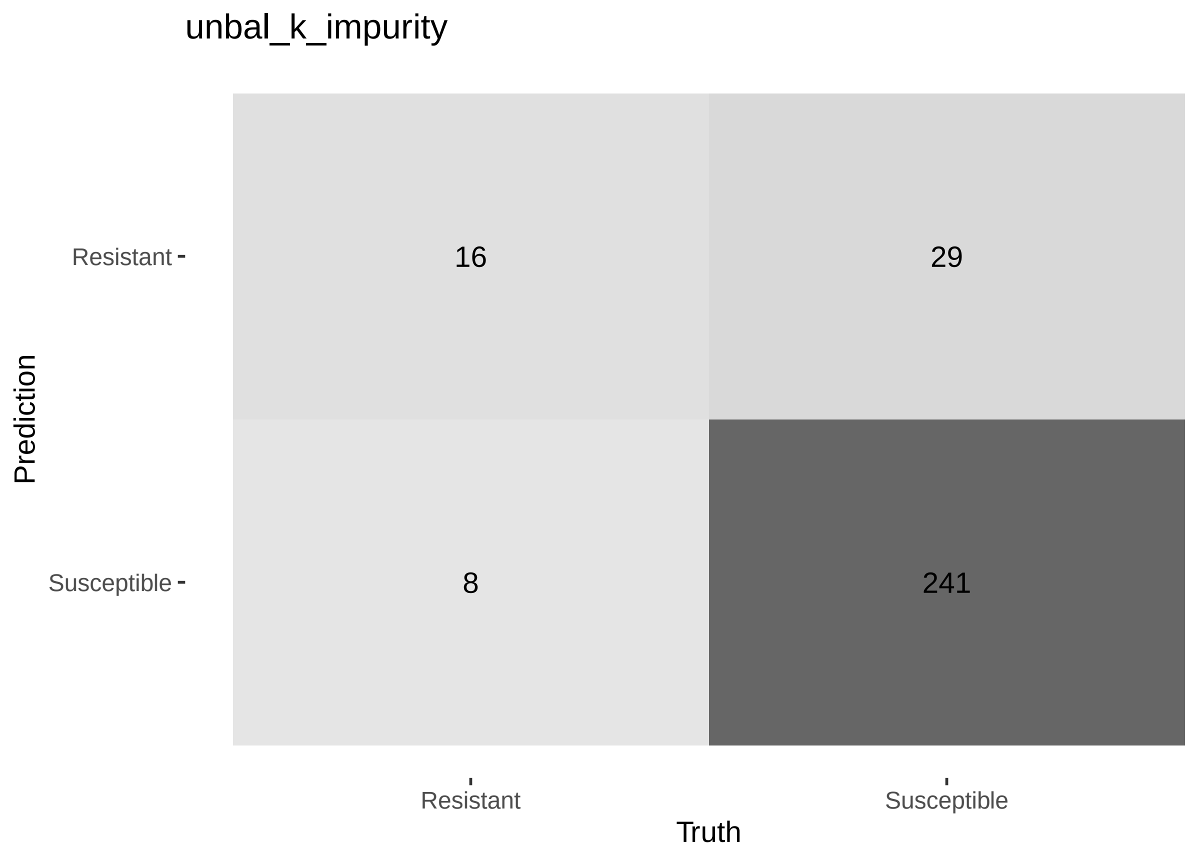


```
auc <- df_roc %>%
     yardstick::roc_auc(truth, pred_resistant)

df_metrics_test <- summary(cm) %>%
    add_row(auc) %>%
    mutate(model = highest_auc)


df_metrics_test %>%
  ggplot(aes(x = .metric, y = .estimate, fill = model)) +
  geom_bar(stat = "identity", position = "dodge") +
  scale_fill_viridis_d() +
  coord_flip() +
  theme_bw()
```

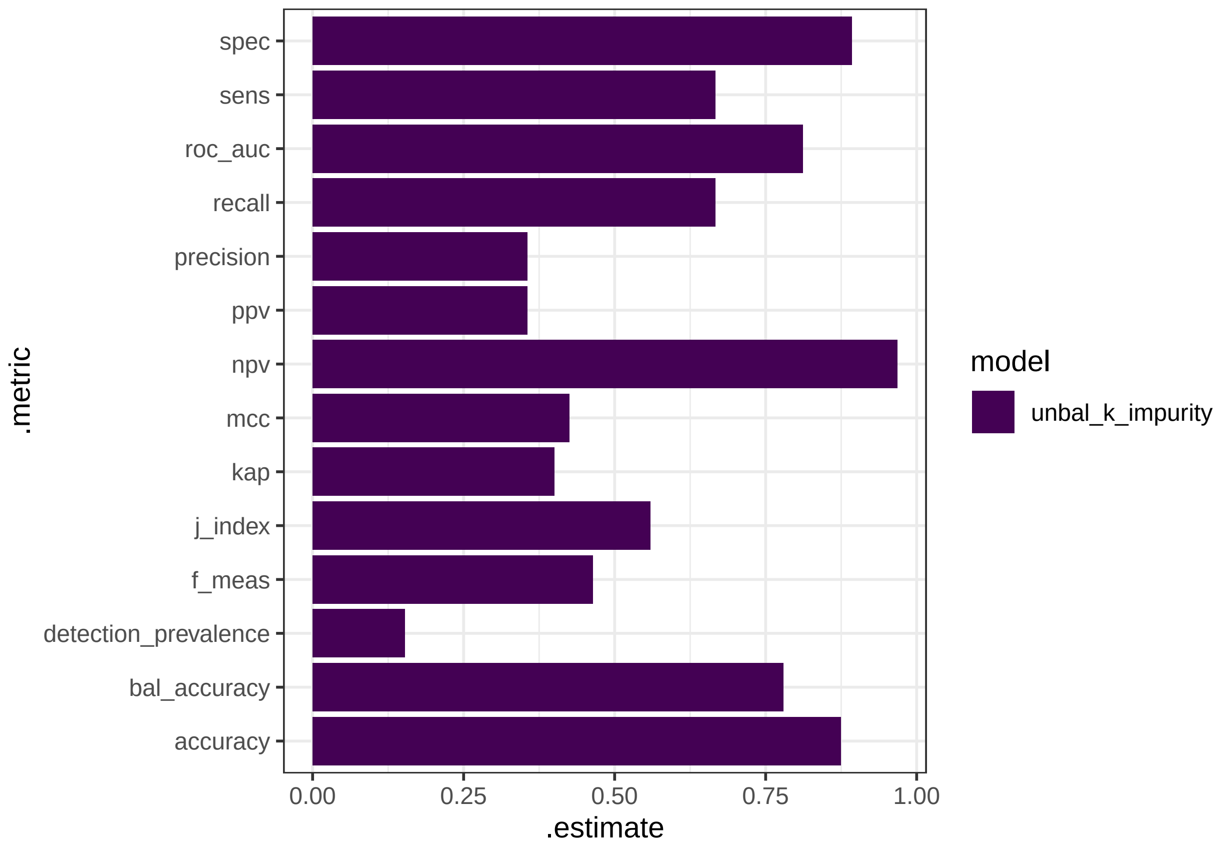


```
# figure with ROC curves merged
auc_train <- df_metrics_train %>%
  filter(model == highest_auc & .metric == "roc_auc") %>%
  .$.estimate
auc_test <- df_metrics_test %>%
  filter(model == highest_auc & .metric == "roc_auc") %>%
  .$.estimate
df_roc_train_test <- df_roc_test %>%
  mutate(y_binary = y_test_binary,
         dataset = "test",
         auc = auc_test) %>%
  bind_rows(df_roc_train %>%
              filter(model == highest_auc) %>%
              mutate(y_binary = y_train_binary,
                     dataset = "train",
                     auc = auc_train))%>%
  mutate(dataset_auc = str_c(dataset, " (AUC: ", round(auc, 2), ")"))


df_roc_train_test %>%
  ggplot() +
  plotROC::geom_roc(aes(d = y_binary, m = pred_resistant, colour = dataset_auc),labels = F) +
 scale_color_manual(values = c("red", "blue")) +
   theme_bw()
```

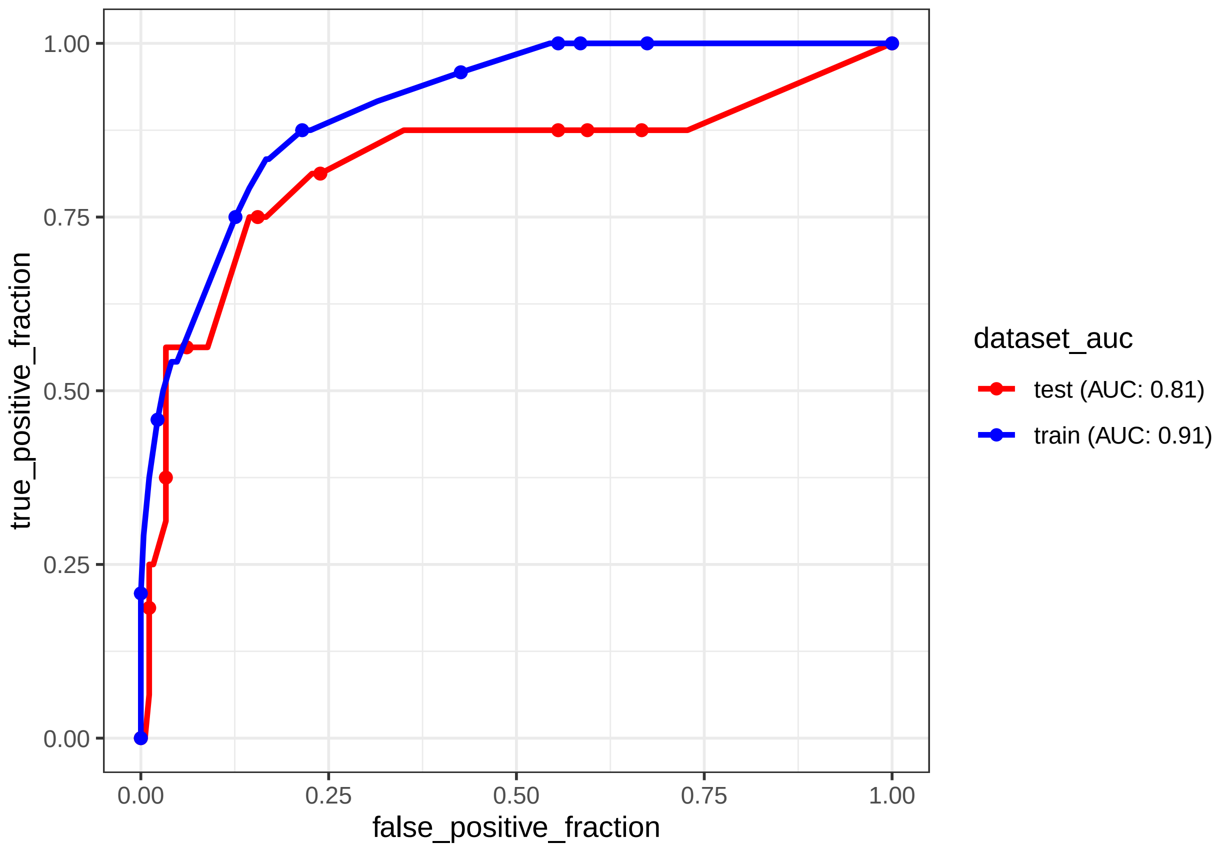

And this is the features plot

```
df_features %>%
  filter(model == highest_auc) %>%
  slice_max(order_by = importance, n = 25) %>%
  ggplot(aes(
    x = fct_reorder(mutation_label, importance),
    y = importance,
    size = af,
    colour = direction
  )) +
  geom_point() +
  scale_colour_manual(values = c(`-` = "blue", `+` = "red"), guide = F) +
  scale_size_continuous(range = c(.5,2),
                        labels = scales::percent,
                        name = "Allele fraction") +
  coord_flip() +
  labs(title = "", x = "", y = "Variable importance")  +
  theme_bw() +
  theme(axis.text = element_text(size = 7, face = "bold"),
        axis.title = element_text(size = 8, face = "bold"),
        legend.title = element_text(size = 7, face = "bold"),
        legend.text = element_text(size = 7, face = "bold"),
        legend.position = "bottom",
        legend.justification = "left")
```

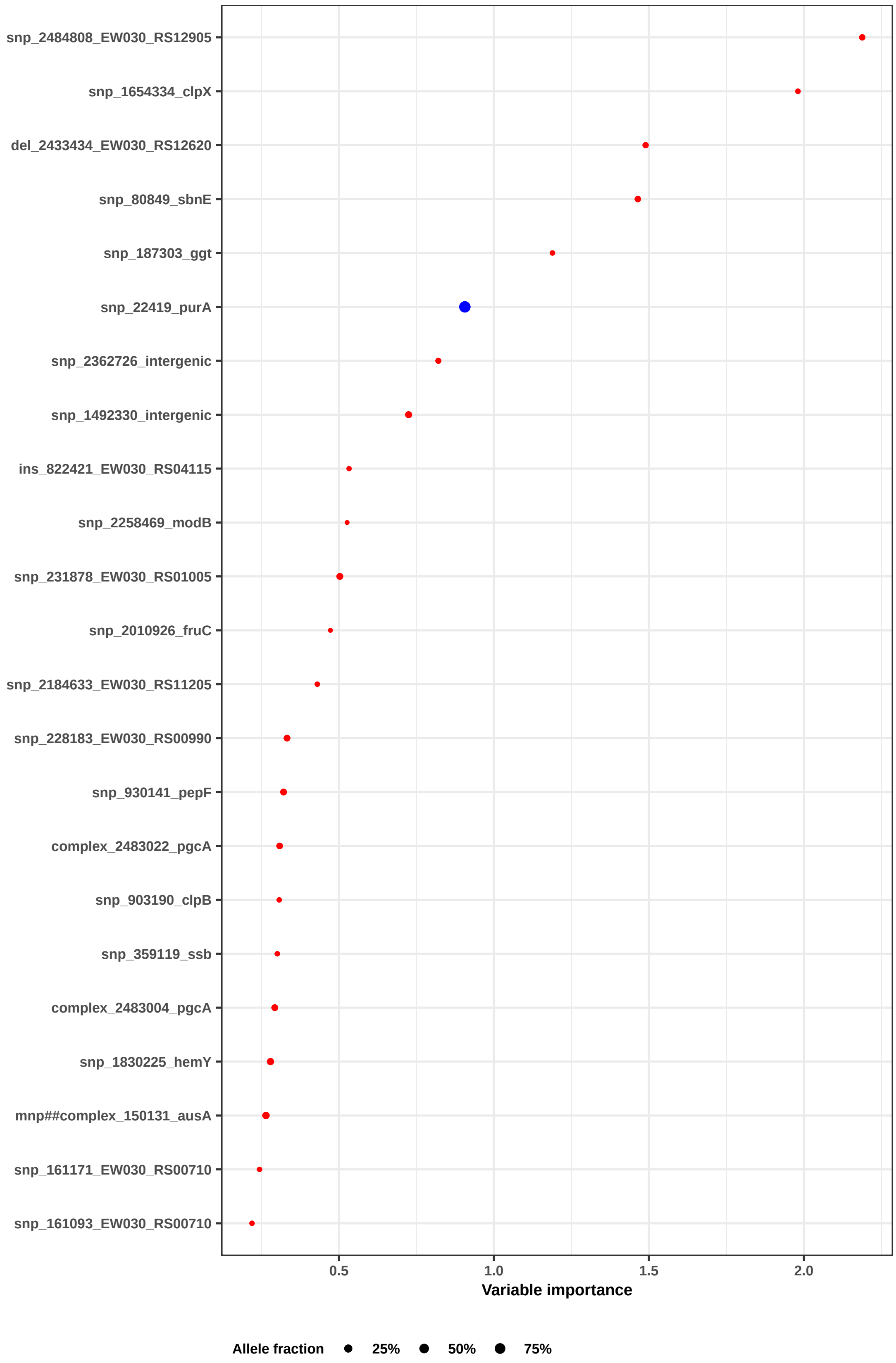
snp_2484808_EW030_RS12905
snp_1654334_clpX
del_2433434_EW030_RS12620
snp_80849_sbnE
snp_187303_ggt
snp_22419_purA
snp_2362726_intergenic
snp_1492330_intergenic
ins_822421_EW030_RS04115
snp_2258469_modB
snp_231878_EW030_RS01005
snp_2010926_fruC
snp_2184633_EW030_RS11205
snp_228183_EW030_RS00990
snp_930141_pepF
complex_2483022_pgcA
snp_903190_clpB
snp_359119_ssb
complex_2483004_pgcA
snp_1830225_hemY
mnp##complex_150131_ausA
snp_161171_EW030_RS00710
snp_161093_EW030_RS00710
0.5
1.0
1.5
2.0
Variable importance
Allele fraction
25%
50%
75%

```r
# ggsave(file = "ranger_95_features_highest_auc_impurity.pdf", width = 3.2, height = 3.8)

# export features file for supplementary data
# df_features %>%
#   filter(model == highest_auc) %>%
#   select(mutation_label, direction, af, or_mixed, p_value_mixed, CHROM, POS, REF, ALT, EFFECT, LOCUS_
#   group_by(mutation_label) %>%
#   slice_head(n=1) %>%
#   arrange(desc(importance)) %>%
#    write_csv("pyseer_binary_mixed_top_mutations_ranger.csv")
```

Of note, clpX is very high and top features have positive direction and a low minimum allele fraction, which is consistent with high specificitt / precision and low sensitivity

Session info

```r
sessionInfo()
```

```
## R version 4.0.2 (2020-06-22)
## Platform: x86_64-apple-darwin17.0 (64-bit)
## Running under: macOS Catalina 10.15.5
##
## Matrix products: default
## BLAS:   /Library/Frameworks/R.framework/Versions/4.0/Resources/lib/libRblas.dylib
## LAPACK: /Library/Frameworks/R.framework/Versions/4.0/Resources/lib/libRlapack.dylib
##
## locale:
## [1] en_AU.UTF-8/en_AU.UTF-8/en_AU.UTF-8/C/en_AU.UTF-8/en_AU.UTF-8
##
## attached base packages:
## [1] stats     graphics  grDevices utils     datasets  methods   base
##
## other attached packages:
##  [1] pROC_1.16.2     yardstick_0.0.7 tictoc_1.0      caret_6.0-86
##  [5] lattice_0.20-41 ranger_0.12.1   magrittr_1.5    forcats_0.5.0
##  [9] stringr_1.4.0   dplyr_1.0.2     purrr_0.3.4     readr_1.3.1
## [13] tidyr_1.1.2     tibble_3.0.3    ggplot2_3.3.2   tidyverse_1.3.0
##
## loaded via a namespace (and not attached):
##  [1] nlme_3.1-149         fs_1.5.0             lubridate_1.7.9
##  [4] httr_1.4.2           tools_4.0.2          backports_1.1.10
##  [7] utf8_1.1.4           R6_2.4.1             rpart_4.1-15
## [10] plotROC_2.2.1        DBI_1.1.0            colorspace_1.4-1
## [13] nnet_7.3-14          withr_2.3.0          tidyselect_1.1.0
## [16] compiler_4.0.2       cli_2.0.2            rvest_0.3.6
## [19] xml2_1.3.2           labeling_0.3         scales_1.1.1
## [22] digest_0.6.25        rmarkdown_2.3        pkgconfig_2.0.3
## [25] htmltools_0.5.0      dbplyr_1.4.4         rlang_0.4.7
## [28] readxl_1.3.1         rstudioapi_0.11      generics_0.0.2
## [31] farver_2.0.3         jsonlite_1.7.1       ModelMetrics_1.2.2.2
```

```
## [34] Matrix_1.2-18       Rcpp_1.0.5          munsell_0.5.0
## [37] fansi_0.4.1         lifecycle_0.2.0     stringi_1.5.3
## [40] yaml_2.2.1          MASS_7.3-53         plyr_1.8.6
## [43] recipes_0.1.13      grid_4.0.2          blob_1.2.1
## [46] crayon_1.3.4        haven_2.3.1         splines_4.0.2
## [49] hms_0.5.3           knitr_1.29          pillar_1.4.6
## [52] reshape2_1.4.4      codetools_0.2-16    stats4_4.0.2
## [55] reprex_0.3.0        glue_1.4.2          evaluate_0.14
## [58] data.table_1.13.0   modelr_0.1.8        vctrs_0.3.4
## [61] foreach_1.5.0       cellranger_1.1.0    gtable_0.3.0
## [64] assertthat_0.2.1    xfun_0.17           gower_0.2.2
## [67] prodlim_2019.11.13  broom_0.7.0         e1071_1.7-3
## [70] class_7.3-17        survival_3.2-3      viridisLite_0.3.0
## [73] timeDate_3043.102   iterators_1.0.12    lava_1.6.7
## [76] ellipsis_0.3.1      ipred_0.9-9
```
